# Characteristic patterns of complement deposition in NMOSD, MOGAD, and MS

**DOI:** 10.1007/s00401-026-02985-9

**Published:** 2026-02-09

**Authors:** Yoshiki Takai, Simon Hametner, Christian Riedl, Tatsuro Misu, Toshiyuki Takahashi, Hiroyoshi Suzuki, Norio Chihara, Masashi Watanabe, Hiroaki Miyahara, Mari Yoshida, Yasushi Iwasaki, Takashi Suzuki, Franziska Di Pauli, Stephan Bramow, Guy Laureys, Brenda Banwell, Sara Mariotto, Kazuo Fujihara, Masashi Aoki, Monika Bradl, Hans Lassmann, Romana Höftberger

**Affiliations:** 1 Department of Neurology, Tohoku University Graduate School of Medicine, Sendai, Miyagi Japan; 2 Department of Pathology, Tohoku University Hospital, Sendai, Miyagi Japan; 3 Division of Neuropathology and Neurochemistry, Department of Neurology and Comprehensive Center for Clinical Neurosciences and Mental Health, Medical University of Vienna, Vienna, Austria; 4 Department of Neurology, National Hospital Organization Yonezawa National Hospital, Yonezawa, Yamagata Japan; 5 Department of Pathology, South Miyagi Medical Center, Shibata, Miyagi Japan; 6 Division of Neurology, Kobe University Graduate School of Medicine, Kobe, Hyogo Japan; 7 Department of Neurology, Ehime Prefectural Central Hospital, Matsuyama, Ehime Japan; 8 Department of Neuropathology, Institute for Medical Science of Aging, Aichi Medical University, Nagakute, Aichi Japan; 9 Department of Neurology, Medical University of Innsbruck, Innsbruck, Austria; 10 Department of Neurology, Danish Multiple Sclerosis Center, Copenhagen University Hospital, Rigshospitalet, Glostrup, Denmark; 11 Department of Neurology, University Hospital Ghent, Ghent, Belgium; 12 Department of Pediatrics, Johns Hopkins University, Baltimore, MD USA; 13 Neurology Unit, Department of Neuroscience, Biomedicine and Movement Sciences, University of Verona, Verona, Italy; 14 Department of Multiple Sclerosis Therapeutics, Fukushima Medical University, Fukushima, Fukushima Japan; 15 Division of Neuroimmunology, Center for Brain Research, Medical University of Vienna, Vienna, Austria

**Keywords:** NMOSD, MOGAD, MS, Complement, C4d, C9neo

## Abstract

The complement system is involved in the pathogenesis of inflammatory demyelinating diseases (IDDs) of the CNS. While complement inhibition significantly reduces the relapse rate in neuromyelitis optica spectrum disorders (NMOSDs), no clear consensus has been reached regarding the role of complement in myelin oligodendrocyte glycoprotein antibody-associated disease (MOGAD) and multiple sclerosis (MS). Therefore, we examined CNS tissues from patients with NMOSD (18 autopsies and one biopsy, median age: 56 years), MOGAD (seven autopsies and 20 biopsies, median age: 34 years) and MS (24 autopsies, median age: 54.5 years) to assess the involvement of the complement system from a histopathological perspective. To investigate complement activity at multiple steps, the tissue deposition of three different complement components (C4d, C3d, and C9neo) was examined using immunohistochemistry. In NMOSD, the typical perivascular rosette/rim pattern of complement deposition was confirmed by the three different complement products within acute astrocyte-lytic lesions. In MOGAD, we observed C4d deposition around perivenous demyelinating lesions in 83% (20/24 tissues). However, C9neo deposition differed between patients, with 73% (11/15 patients with perivenous demyelination-predominant MOGAD) showing limited deposition of C9neo with relatively well-preserved oligodendrocytes (MOGAD type A), while 27% showing strong deposition accompanied by the disappearance of oligodendrocytes (MOGAD type B). The more destructive type B pathology was more frequent among deceased than living patients who, by contrast, had type A pathology in the vast majority. In MS, only C4d showed clear deposits on myelin sheaths in the peri-plaque white matter bordering the edges of the demyelinating lesions. These findings seemed to be characteristic of MS, and the extent and intensity tended to decrease in accordance with lesion activity. Complement deposition in MS lesions was linked to shorter interval between onset and death. These characteristic patterns of complement deposition in the three IDDs likely reflect the distinct pathogeneses of the diseases.

**Supplementary Information:**

The online version contains supplementary material available at 10.1007/s00401-026-02985-9.

## Introduction

Complement activation is an important mechanism of immune defence against foreign pathogens, but it may also drive tissue injury in autoimmune conditions. It involves the sequential activation and cleavage of different proteins, ultimately leading to the assembly of the C_5–9_ (C9neo) complex, which has a cytotoxic function. However, other components of this cascade have additional functions, even in the absence of terminal complement complex formation [33]. These functions include the proinflammatory functions of C3a and C5a and the opsonization of targets by C3 cleavage products, which promote phagocytosis and antibody-dependent cellular cytotoxicity [41]. Although the function of C4d has yet to be identified, C4d covalently binds with high affinity to tissue constituents where complement activation takes place; C4d binds to tissue constituents with a greater affinity than other complement components. Thus, C4d is regarded as a sensitive marker of complement activation in immunological diseases [28]. In the brain, many components of the complement system are locally produced by neurons and glia, and they have multiple functions in health and disease [5]. During plasticity, CNS injury and repair, their local production is upregulated, which promotes the phagocytosis of cells and their processes [25].

The complement system is a crucial factor in inflammatory demyelinating diseases (IDDs) of the CNS. In recent years, many biological agents have been developed, confirming the importance of therapeutic strategies based on the key pathophysiological changes associated with each of these diseases. In particular, in AQP4 antibody-positive neuromyelitis optica spectrum disorders (AQP4 + NMOSDs), limiting complement activity has a strong ability to prevent relapse [34, 35]. One important explanation for this effect is the evidence of active complement deposition around blood vessels in AQP4 + NMOSDs [22, 26, 39].

However, the involvement of the complement system in the pathogenesis of other IDDs remains unclear. The expression of the downstream complement component C9neo, which reflects membrane attack complex (MAC) formation, was reported in so-called Pattern 2 lesions in a subset of patients with early multiple sclerosis (MS) [21]. In contrast, deposition of upstream complement components (C1 and C3d) was almost universally found in active lesions in a cohort dominated by patients with long-lasting progressive disease [3]. It remains unclear whether complement activation varies only between individuals with MS or also across disease stages within individuals. MOG antibody-associated disease (MOGAD) was recently defined following the discovery of conformation-sensitive MOG antibodies [2], but research on the involvement of the complement system in MOGAD has been inconsistent and has not provided a clear answer [11, 38]. Most importantly, however, clarifying complement deposition patterns across inflammatory demyelinating conditions could improve the collaboration between neuropathologists and neurologists who diagnose NMOSD, MOGAD, MS and other conditions involving white matter injury.

Therefore, we performed a pathological study, analysing different components of complement activation in a large set of well-characterized human inflammatory demyelinating diseases, including NMOSD, MOGAD and MS. By using tissues from patients with septic meningoencephalitis, tumours, and ischaemic conditions as controls, we identified characteristic complement deposition patterns in each IDD and confirmed the differences among the pathophysiological changes associated with the different diseases.

## Materials and methods

### Patients

We analysed CNS tissues from 18 autopsied patients and one biopsied patient with NMOSD, 24 autopsied patients with MS, and seven autopsied and 20 biopsied patients with MOGAD (two autopsied and four biopsied patients included in a study by Höftberger et al. 2020 [11]; one autopsies patient included in a study by Carta et al. 2021 [4]; nine biopsied patients included in a study by Takai et al. 2020 [38]). For 6/7 autopsied MOGAD patients and 9/20 MOGAD biopsied patients with perivenous demyelinating lesions, there was enough remaining tissue for further classification of complement deposition patterns. In 14 autopsied patients and one biopsied patient with a diagnosis of NMOSD from whom tissues were obtained between 2010 and 2024, anti-AQP4 antibodies (AQP4-Ab) were detected in the serum by a live cell-based assay (CBA). The remaining four patients with unknown AQP4-Ab status, from whom tissues were obtained between 1989 and 2001, were clinically diagnosed with NMOSD and presented astrocytopathic lesions with AQP4 loss [26, 36] and characteristic complement deposition [39] upon histopathological analysis. In all patients with MOGAD, the presence of anti-MOG antibodies (MOG-Ab) was confirmed by a live CBA (full-length MOG), and all of these patients were retrospectively confirmed to meet the criteria proposed by the international MOGAD panel [2]. Brain biopsies were performed between 2004 and 2019, and autopsies were performed between 2015 and 2023. The two patients in whom MOG-Ab could not be detected at the time of brain biopsy (performed in 2004 and 2008) were subsequently found to be positive for MOG-Ab. Among patients with MS, the status of AQP4-Ab and MOG-Ab was unavailable for all cases except for one patient confirmed to be negative for AQP4-Ab. Six patients with atypical demyelinating lesions were excluded from the analysis: one with mainly perivenous demyelination (PVD), two with concentric demyelination, and three with severe necrotic tissue damage. Of the remaining 18 cases, 17 had been clinically diagnosed with MS and showed focal or confluent, sharply demarcated inflammatory demyelinating lesions consistent with MS. The remaining case was a patient who died as a result of a traffic accident and who had no clinical diagnosis or documented symptoms suggestive of MS during his lifetime. However, inflammatory demyelinating lesions with pathological features typical of MS, including the presence of a mixed active/inactive demyelinating lesion, were identified at autopsy, and this case was therefore included in the analysis. The analyzed patients with MS were autopsied between 1978 and 2022. Characteristics of clinical presentation and brief pathological findings for each disease are summarized in Table 1, and data for individual cases are provided in Supplementary Table 1. The cohort of NMOSD patients included 16 women and three men, with a median age of 56 years (range 20–87 years). The MOGAD cohort included 13 women and 14 men, with a median age of 33.5 years (range 4–81 years). The MS cohort included 11 women and 7 men, with a median age of 56 years (range 34–79 years). Treatment data are only listed for immunotherapies administered within approximately 6 months prior to the biopsy or autopsy. To control for neurological disease and other types of brain lesions, we also analysed patients with acute cerebral infarction with or without brain haemorrhage (n = 7), chronic cerebral infarction (n = 5), brain haemorrhage (n = 1), septic meningoencephalitis (n = 3), glioblastoma (n = 5), and malignant lymphoma (n = 4) and normal controls (n = 5).

**Table 1:** Clinical and pathological characteristics of inflammatory demyelinating diseases

		NMOSD	MOGAD	MS
Patients (no)	Autopsy	18	7	18
	Biopsy	1	20	0
Age at biopsy/autopsy (yr), median (range)		56 (20–87)	33.5 (4–81)	54.5 (28–79)
Sex (F/M)		16/3	13/14	11/7
Disease duration (mo), median (range)		34 (0.6–252)	2 (0.1–168)	276 (16–504)
Interval between the last relapse and biopsy/autopsy (mo), median (range)		3 (0.4–108)	1 (0.1–98)	138 (5–216)
Disease course	Monophasic	6/18	19/27	0/18
	Relapsing	12/18	7/27	5/18
	Progressive	0/18	1/27	13/18
Disease state at biopsy/autopsy	Acute phase	12/18	26/27	0/18
	Chronic phase	6/18	1/27	18/18
Treatment				
Immunotherapy-naïve		1/18	13/25	6/11
Acute-phase therapy	IVMP	12/12	11/24	-
	PE	3/12	3/24	-
	IVIG	0/12	2/24	-
Conventional immunosuppressants*		4/18	1/25	2/11
Non-MS targeted biologics**		1/18	2/25	0/11
MS-DMTs***		0/18	0/25	3/11
Tissue blocks characteristics				
Regional distribution	Brain	7	37	29
(number of tissue blocks)	Brainstem	12	5	11
	Spinal cord	37	3	4
	Optic nerve	1	1	3
Lesion stage of analysed tissue blocks	Acute	19/57	37/46	7/47
	Subacute	11/57	17/46	19/47
	Chronic	33/57	6/46	32/47

^*^Conventional immunosuppressants include azathioprine, cyclosporine, cyclophosphamide and tacrolimus;

^**^Non-MS targeted biologics include satralizumab, rituximab, and tocilizumab;

^**^MS-DMTs include interferon beta and fingolimod

*F* female, *M* male, *MS* multiple sclerosis, *NA* not applicable, *NMOSD* neuromyelitis optica spectrum disorders, *no* number, *mo* month, *MOGAD* myelin oligodendrocyte glycoprotein antibody- associated disease, *MS* multiple sclerosis, *MS-DMTs* MS disease-modifying therapies, *yr* year

### Neuropathological examinations and immunohistochemistry

For neuropathological examinations, 2–4 μm thick slices of paraffin-embedded biopsied or autopsied tissues were evaluated using standard neuropathological methods, including haematoxylin and eosin (H&E) staining, Klüver–Barrera (KLB) myelin staining, and immunohistochemistry. Bright-field immunohistochemistry was performed either manually or using the automated platform Autostainer Link 48 (Dako). The primary antibodies and protocols used for immunohistochemistry in this study are listed in supplementary Table 2. Briefly, the paraffin-embedded sections were deparaffinized for 2 × 10 min in xylene and rehydrated. Endogenous peroxidase activity was subsequently blocked by the addition of 3% H_2_O_2_ in methanol for 10 min. After the samples were rinsed with TBS, heat-induced antigen retrieval was performed using a PT Link water bath (Dako) at 95 °C for 20 min, after which either citrate buffer (pH 6.0) or EDTA buffer (pH 9.0) was applied. Alternatively, proteinase K proteolytic digestion for 5 min at room temperature (RT) was performed, depending on the primary antibody (see Supplementary Table 2). After being rinsed with deionized water and blocking unspecific antibody binding with 10% foetal calf serum in TBS for 20 min, the slides were incubated with primary antibodies either at 4 °C overnight (manual staining) or for 30 min at room temperature using the automated platform Autostainer Link 48 (Dako). After the slides were washed with TBS, the K8002 (Dako EnVision Flex + detection system) or K5007 (Dako Real EnVision HRP) secondary system was used according to the manufacturer’s protocols. To visualize the staining, diaminobenzidine hydrochloride (DAB) chromogen solution was applied for 10 min at RT. After counterstaining, all glass slides were coverslipped, and high-resolution digital images were obtained with a slide scanner (NanoZoomer-SQ, Hamamatsu). Quantitative evaluation was performed using a dedicated viewing software (NDP.view2, Hamamatsu).

### Immunohistochemistry enzyme double staining

Following deparaffinization and rehydration, heat-induced antigen retrieval was performed in a steamer using the “Diva Decloaker” (Biocare Medical, Concord, CA). After blocking unspecific binding with 10% goat serum, the slides were incubated with primary antibodies at 4 °C overnight. After the slides were washed three times with PBS, endogenous peroxidase activity was blocked by incubation in 30% methanol/PBS containing 1% H_2_O_2_ for 20 min. After the slides were further washed with PBS, alkaline phosphatase (AP)-conjugated secondary antibodies (Histofine Simple Stain kit [Nichirei, Tokyo]) were applied to the slides, which were subsequently incubated at RT for 40 min. After staining the target blue using the Vector Blue AP Substrate Kit, the slides were incubated with the second primary antibody at 4 °C overnight. After the slides were washed, HRP-conjugated secondary antibodies (Histofine Simple Stain kit [Nichirei, Tokyo]) were applied at RT for 40 min. To stain the second primary antibody target brown, the DAB chromogen solution was applied for 5 min at RT. After nuclear staining with Nuclear Fast Red (Vector), all the slides were sealed and coverslipped using VectaMount (Vector).

### Immunofluorescence double staining of MOG and C4d

Following deparaffinization, rehydration and blocking of endogenous peroxidase activity as described above, citrate buffer (pH 6.0) was used for antigen retrieval in a steamer. Paraffin autofluorescence was blocked using 1% sodium borohydride in TBS for two minutes twice. Unspecific binding of antibodies was blocked by incubation in 10% normal goat serum for 30 min. Primary antibodies (MOG 1:125, C4d 1:50; the same antibodies used for single staining as described above) were applied simultaneously in Dako antibody diluent. Following overnight incubation at 4°C, fluorophore-conjugated anti-rabbit secondary antibodies conjugated to Alexa Fluor 488 (1:800, #711–485-152, Jackson ImmunoResearch) and anti-mouse secondary antibodies conjugated to Cy3 (1:1000, #115–165-166, Jackson ImmunoResearch) were applied in Dako antibody diluent for 1.5 h at RT. After nuclear staining with 1 µg/ml 4,6-diamidino-2-phenylindole (DAPI; D1306, Invitrogen) in TBS, the slides were coverslipped using aqueous mounting medium (18,606, Polysciences) and stored at 4°C. Images were taken using a fluorescence microscope (BX63, Olympus) and Olympus CellSens software.

### Staging of NMOSD lesions

All NMOSD lesions were classified as one of the following four astrocytopathy-based stages, as previously reported [39].

(A) Astrocyte lysis stage: acute lesions; the earliest stage of NMOSD lesions and characterized by extensive loss of astrocytes with or without fragmented and/or dust-like particles of astrocytes.

(B) Progenitor recruitment stage: acute to subacute lesions; characterized by widespread disappearance of mature astrocytes but the presence of small nucleated cells with GFAP-positive unipolar or bipolar foot processes.

(C) Protoplasmic gliosis stage: subacute to chronic lesions; characterized by the presence of GFAP-positive star-shaped astrocytes, minority (< 50%) of which are attached to each other via foot processes.

(D) Fibrous gliosis stage: chronic lesions; composed of densely packed mature astrocytes.

For evaluation, lesions featuring astrocyte lysis were considered acute stage lesions, lesions featuring progenitor recruitment were considered subacute lesions, and lesions featuring protoplasmic gliosis and fibrous gliosis were pooled together as chronic stage lesions in this study.

### Staging of inflammatory demyelinating lesions

Demyelinating lesions were classified based on the severity of demyelination, as previously reported [16, 19].

1. Active and demyelinating lesions: lesions showing ongoing demyelination, characterized by myelin loss and dense infiltrates of phagocytic macrophages containing myelin degradation products in their cytoplasm (LFB + or MBP + or PLP + myelin degradation products) throughout the lesion area.
2. Active and postdemyelinating lesions: demyelinated lesions with macrophages lacking myelin degradation products in their cytoplasm (PAS + myelin degradation products may still be detectable) throughout the lesion area.
3. Mixed active/inactive lesions: demyelinated lesions characterized by a hypocellular lesion centre and a rim of activated macrophages containing myelin degradation products at the lesion edge.
4. Inactive demyelinated lesions: demyelinated lesions with little or no macrophage infiltration and without remyelination.
5. Inactive and fully or mostly remyelinated lesions (shadow plaques): sharply demarcated lesions with uniformly thin and less dense myelin than normal white matter.

In this study, active and demyelinating lesions and active and postdemyelinating lesions were pooled as active lesions.

### Evaluation of the complement deposition rate in NMOSD lesions at each stage

Lesions in tissues in each block were staged based on astrocytopathy; if lesions of different stages were present in the same block, they were evaluated as separate lesions. The deposition of C4d, C3d, and C9neo in each lesion was classified into either a rosette pattern or a rim pattern. If each complement product was clearly stained around blood vessels and three or more vessels were identified in the same block, it was evaluated as definite deposition. If two or fewer complement products were identified or if complement staining was weak and deposition was observed only in parts of the perivascular tissue, it was considered mild deposition. The positivity rate was calculated as the ratio of the number of blocks containing complement-deposited lesions to the total number of blocks containing lesions.

### Comparison of the number of vessels with perivascular complement deposition between MOGAD and NMOSD patients

To compare the number of vessels with complement deposition between NMOSD and MOGAD patients, we counted vessels with complement deposition in the rim and rosette patterns in NMOSD patients and vessels with complement deposition accompanied by perivenous demyelination in MOGAD patients. Capillary vessels with a diameter of 10 µm or less were not counted. In each tissue, depending on the size of the lesion, 3–4 circular regions of 1.25–2.5 mm^2^ (totalling between 3.75 and 10 mm^2^) were demarcated such that the number of vessels with complement deposition was maximized. The deposition of C4d, C3d, and C9neo was evaluated, and the density of complement-deposited vessels was calculated as the number per mm^2^. To compare the deposition frequency for each complement component (C4d, C3d, and C9neo), we calculated the C3d/C4d ratio and C9neo/C4d ratio by dividing the number of vessels with C3d or C9neo deposition by the number of vessels with C4d deposition in each lesion.

### Assessment of the TPPP-positive oligodendrocyte density within demyelinating lesions

We counted the number of TPPP-positive cells present within each demyelinating lesion. For each lesion, three to five circular regions of 0.125–1.0 mm^2^ were randomly demarcated (0.5–5 mm^2^), depending on the size of the lesion, and the density of TPPP-positive cells was calculated as the number per mm^2^.

### Grading of the extent of complement deposition in the perilesional pattern in MS

Complement deposition at the periphery of confluent demyelinating lesions in MS was classified as follows based on the extent and intensity of C4d staining.

All: C4d deposited in more than 70% of the entire peripheral area of the demyelinating lesion.

Half: C4d deposited in 30–70% of the entire peripheral area of the demyelinating lesion.

Partial: C4d deposited in less than 30% of the entire peripheral area of the demyelinating lesion.

No: No C4d deposition.

In MS patients, demyelinating lesions were evaluated as the same lesion when they were fused and as different when they were separate. We classified all the lesions based on the MS stage and evaluated the degree of complement deposition in each stage. For comparative analysis of disease duration and complement deposition among MS patients, deposition grades of all, half, or partial for C4d were considered to indicate C4d deposition.

### Statistical analysis

Student’s *t* test was used to compare the intervals from disease onset to death between the two groups based on the presence or absence of complement deposition in MS lesions. To compare the number of complement-deposited vessels in NMOSD and MOGAD type-specific lesions among the three groups, the Tukey–Kramer method was used. *P* values < 0.05 were considered to indicate a statistically significant difference.

### Data availability

The datasets that support the findings of the current study are available from the corresponding author upon reasonable request. The data are not publicly available because they contain information that could compromise the privacy of the research participants.

## Results

### Characteristics of complement deposition patterns in each IDD

#### NMOSD

##### Clinical characteristics and treatment status of the NMOSD cohort

Detailed clinical information was available for 18 of the 19 patients included in the study. The median disease duration was 34 months (range, 0.6–252 months), and the median interval between the last clinical relapse and biopsy or autopsy was 3 months (range, 0.4–108 months). Regarding disease course, 6 of the 18 patients (33%) exhibited a monophasic course, whereas 12 patients (67%) had a relapsing course; none of the patients showed a progressive course. At the time of biopsy or autopsy, 12 patients (67%) were classified as being in the acute phase, while the remaining 6 patients (33%) were in the chronic phase. With respect to treatment status, one patient (1/18, 6%) was immunotherapy-naïve. Among the 12 patients in the acute phase, all received intravenous methylprednisolone (IVMP) as acute-phase therapy, with plasma exchange (PE) additionally administered in 3 patients. Beyond acute-phase management, conventional immunosuppressive agents were used in 4 patients, including azathioprine (n = 1), cyclosporine (n = 2), and tacrolimus (n = 1). In addition, one patient was treated with satralizumab.

##### Patterns of complement deposition in NMOSD

**Fig. 1:**
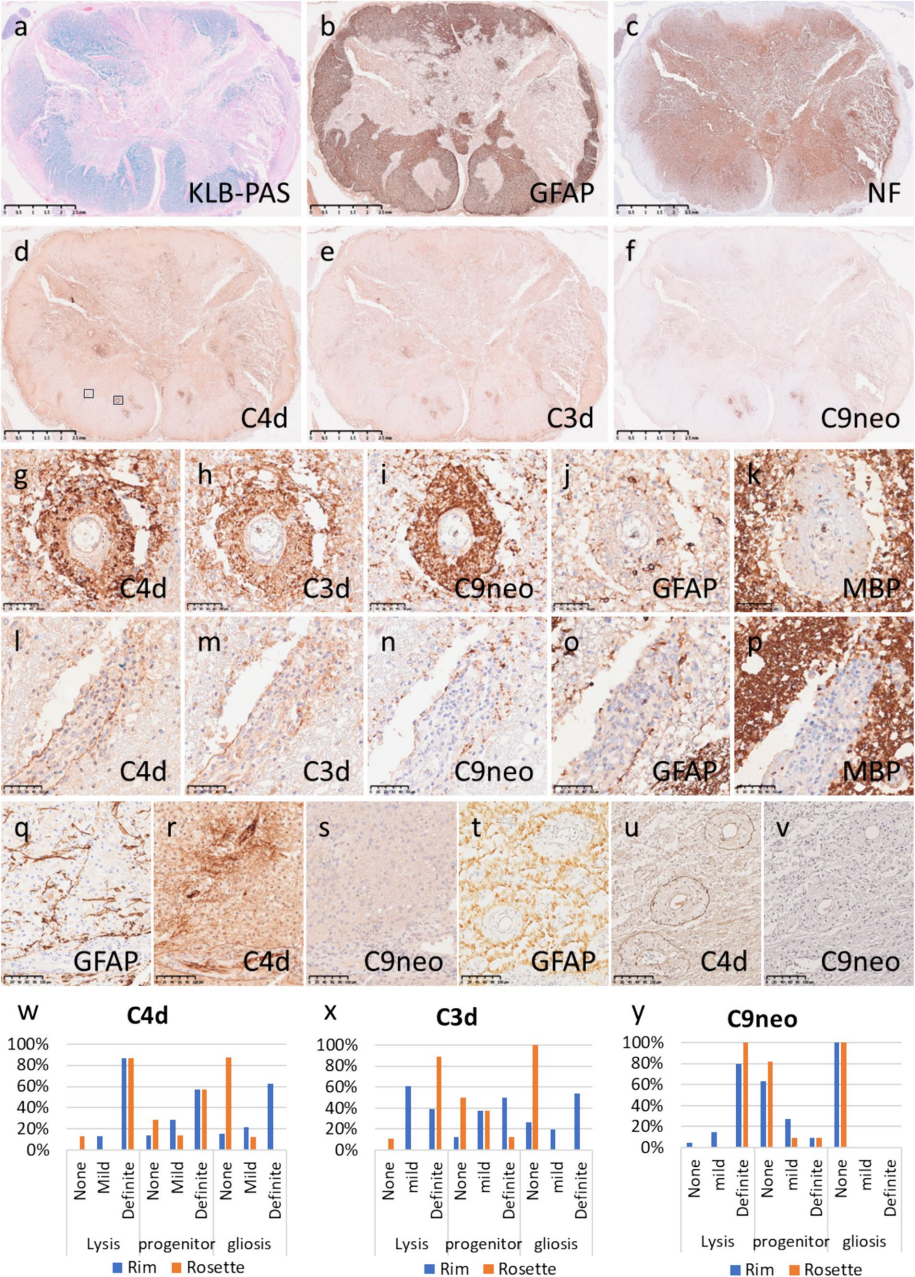
Complement deposition patterns in NMOSD patients. **a‒p** Acute NMOSD lesions in the spinal cord (NMO-a15). Active demyelinating lesions (**a**, KLB-PAS) with extensive astrocytic destruction (b, GFAP) were observed in the spinal cord. The axons were relatively preserved (**c**, NF). C4d (d, g and l), C3d (e, h and m), and C9neo (**f, i** and **n**) showed similar staining patterns, with a rosette pattern (g–i) and a rim pattern (l–n) being characteristic of NMOSD. Such findings were particularly prominent in the acute NMOSD lesions, in which astrocytes were destroyed (j and o, GFAP), but the myelin sheaths were relatively preserved (k and p, MBP). q–s Subacute NMOSD lesions in the brain (NMO-b1). Astrocyte progenitor cells were observed, and the tissue showed partial gliosis (q, GFAP). C4d was deposited around blood vessels, showing both a rosette pattern and a rim pattern (r), whereas C9neo was observed only as small dot-like deposits scattered around the blood vessels (s). t–v Chronic NMOSD lesions in the spinal cord (NMO-a13). Astrocytes had regenerated and showed fibrous gliosis (t, GFAP). C4d was deposited in a rim pattern around blood vessels (u), but deposition of C9neo was not observed (v). w‒y Comparison of the deposition patterns of each complement pathway product and the stage of astrocytopathy. The rosette pattern (red bar) was characteristic of the acute stage (from the lysis stage to the progenitor stage) and was rarely seen in the chronic gliosis stage, for any of the complements (w, C4d; x, C3d; y, C9neo). However, the rim pattern (blue bar) could be seen in all stages, except for staining with C9neo (w–y). Scale bars: a–f = 2.5 mm; g–p = 50 µm; q–v = 100 µm. *GFAP* glial fibrillary acidic protein, *KLB-PAS* Klüver–Barrera periodic acid–Schiff, *MBP* myelin basic protein, *NF* neurofilament, *NMOSD* neuromyelitis optica spectrum disorder

First, we evaluated complement deposition patterns in NMOSD lesions, which have already been extensively reported. Fifty-six tissue blocks (6 forebrain, 12 brainstem, 37 spinal cord, and 1 optic nerve tissue blocks) from 18 autopsied patients (12 AQP4 antibody positive, 6 untested) and one biopsied brain tissue block from an AQP4 + NMOSD patient were analysed (Table 1). In acute lesions with extensive astrocyte destruction but some preservation of the myelin sheaths and axons (Fig. 1a–c), complement deposition was observed in a mesh-like pattern around vessels (rosette pattern) (Fig. 1d–k) or along the glia limitans (rim pattern) (Fig. 1l–p). Staining along the meninges was also identified as a subtype of the rim pattern (Fig. 1d–f). Rosette patterns were almost always found within the astrocyte-destructing lesion, but a rim/pia pattern of deposition, especially for C3d/C4d, was also observed where astrocytes were present (Fig. 1q–v). Many deposits were observed in the subacute to chronic phase (progenitor recruitment stage–fibrous gliosis stage) adjacent to regenerating astrocytes (Fig. 1w–y). In acute astrocyte-destructive lesions, complement deposition was also observed on the cell bodies of astrocytes and their fragments (Supplemental Fig.1).

##### Relationship between the type of deposited complement pathway product and temporal variation in NMOSD

The characteristics of complement deposition in NMOSD varied depending on the stage of the lesion. In the acute stage, the rosette pattern was predominant, but the rim pattern was also observed, and the staining patterns of C4d, C3d and C9neo were similar (Fig. 1 g–i, l–n and w–y). C9neo deposition became scarce from the subacute stage onwards (Fig. 1s and y) and was almost absent in the chronic stage (Fig. 1v and y). C4d and C3d staining patterns were similar and clearly visible from the subacute stage to the chronic stage (Fig. 1r, u, w and x), but the rosette pattern became less obvious as the lesion stage progressed; only the rim pattern was seen almost exclusively in the chronic stage (Fig. 1w–x).

#### MOGAD

##### Clinical characteristics and treatment status of the MOGAD cohort

Clinical information was available for all 27 patients included in the study, whereas detailed treatment data were available for 25 patients. The median disease duration was 2 months (range, 0.1–168 months), and the median interval between the last clinical relapse and biopsy or autopsy was 1 month (range, 0.1–98 months). With respect to disease course, classifications were based on the clinical course at the time of tissue acquisition in biopsy cases. Nineteen of the 27 patients (70%) exhibited a monophasic course, 7 patients (26%) had a relapsing course, and 1 patient (4%) showed a progressive course. At the time of biopsy or autopsy, 26 patients (96%) were classified as being in the acute phase, whereas one patient (4%) was in the chronic phase. Regarding treatment status, 13 of the 25 patients (52%) with available treatment data were immunotherapy-naïve. Among the 24 patients classified as being in the acute phase, IVMP was administered in 11 patients, PE in 3 patients, and intravenous immunoglobulin in 2 patients. Cyclophosphamide pulse therapy and rituximab were each used as acute-phase treatment in one patient, whereas tocilizumab was administered as relapse-prevention therapy in one patient.

##### Patterns of complement deposition in MOGAD

**Fig. 2:**
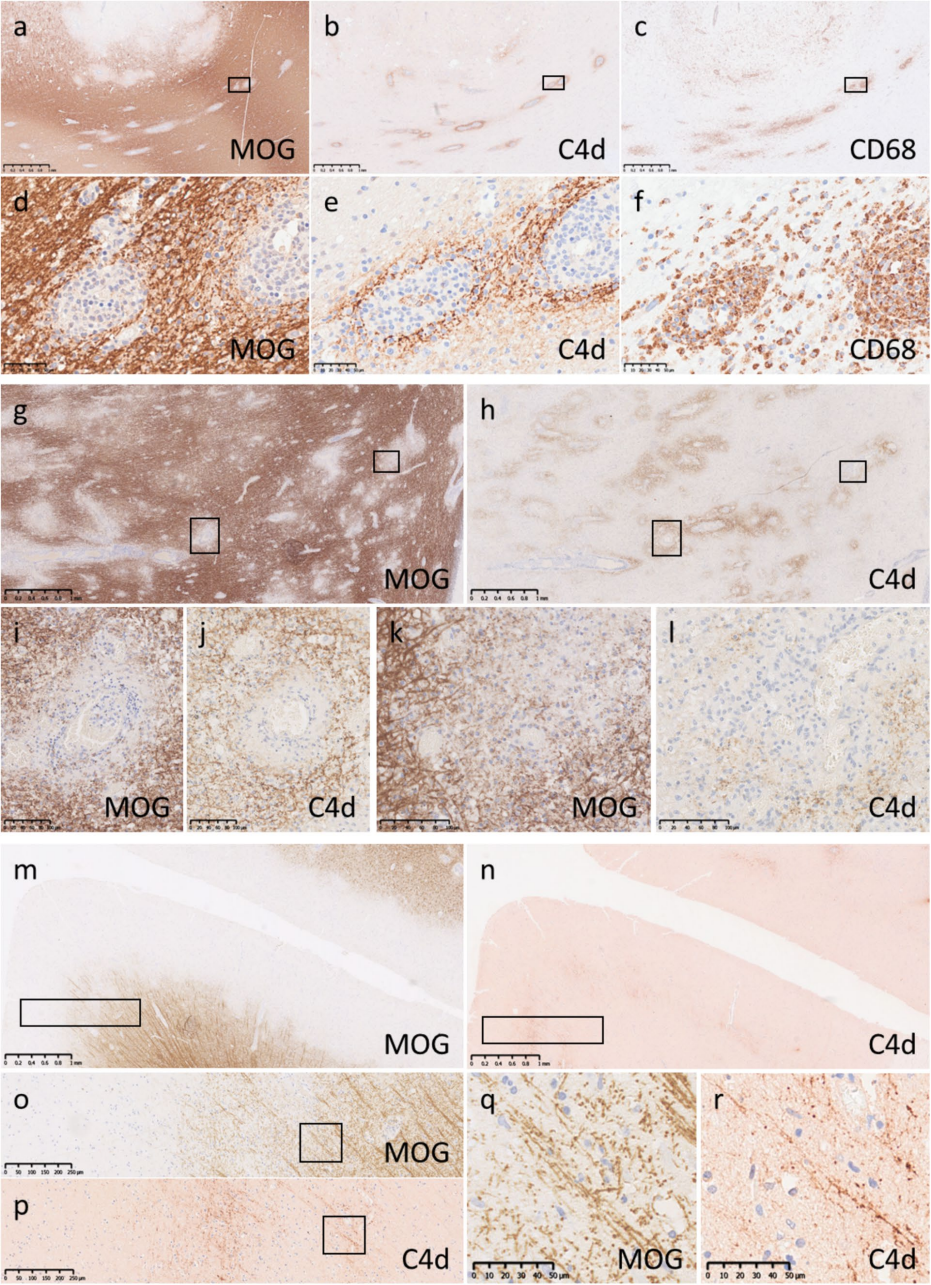
Complement deposition patterns in MOGAD. a–f Brain (MOG-a6). Along with intracortical demyelinating lesions, multiple typical perivenous demyelinating lesions were observed from the corticomedullary junction to the white matter (a and d, MOG). C4d deposition (b and e, C4d) and macrophage infiltration (c and f, CD68) were seen consistent with perivenous demyelinating lesions. d–f Magnified images of the boxed area in panels a–c. C4d was deposited on the myelin sheath (e, C4d) at the site of perivenous demyelination (d, MOG). Many macrophages infiltrated perivenous demyelinating lesions but were predominantly present in the perivascular space (f, CD68). g–l Cerebellum (MOG-a4). Numerous perivenous demyelinating lesions were observed, some of which fused to form large demyelinating foci (g, MOG). C4d was consistently deposited at the edge of the demyelinating lesions (h, C4d). i–j and k–l Magnified images of the boxed areas in the lower left and upper right of panels g and h. C4d was not deposited in areas where MOG had been lost due to perivenous demyelination (i, MOG) but was bound to the myelin sheath, which was slightly distant from the glial limitans (j, C4d). In demyelinating lesions with fused perivenous demyelination (k, MOG), C4d was deposited on the myelin sheath at the margins of the lesion (l, C4d). m–r Brain (MOG-a3). The presence of subpial demyelinating lesions was confirmed by MOG staining (m, MOG). C4d was deposited at the border of the demyelinated area (n, C4d). o and p Magnified images of the boxed areas in panels m and n. q and r Magnified images of the boxed areas in panels o and p. C4d (p and r, C4d) was deposited on the remaining myelin sheath (o and q, MOG) at the border of demyelinating lesions. Scale bars: a–c, g–i and m–n = 1.0 mm; d–f and a–r = 50 µm; i–l = 100 µm; o–p = 250 µm. *MOG* myelin oligodendrocyte glycoprotein.

Complement deposition in MOGAD was examined using 23 tissue blocks from 7 autopsied patients (14 forebrain, 5 brainstem, 3 spinal cord, and 1 optic nerve tissue blocks) and 23 tissue blocks from 20 biopsied patients (all forebrain tissue blocks). Twenty-eight tissues (from 6 autopsied patients and 12 biopsied patients) exhibited mainly PVD combined with various degrees of fused lesions or subpial demyelination. Most of these tissues showed deposition of the complement split product C4d, except for four samples that could not be analysed due to insufficient material. C4d deposition was observed from the border of PVD to the surrounding myelinated area in 83% (20/24) of these samples (Fig. 2a–f). In the early phase of PVD, C4d was deposited on myelin sheaths close to the perivascular glia limitans, with the perivascular myelin sheaths still preserved (Fig. 2d and e). For lesions with more advanced PVD and myelin sheaths that had disappeared from the perivascular brain parenchyma, C4d labelled myelin at the border of the demyelinated tissue (Fig. 2g–j), which was also confirmed in the large demyelinating lesions caused by fused PVD (Fig. 2g–h and k–l). Fifteen tissues (from 2 autopsied patients and 6 biopsied patients) exhibited predominantly confluent demyelination; C4d deposition was observed at the edge of demyelinating lesions as described previously in 73.3% of these tissues, and only one tissue exhibited C4d deposition around the vessels located inside the confluent demyelination zone. Furthermore, in 60% (9/15) of the samples, C4d was deposited on the remaining myelin sheaths during confluent demyelination (Supplemental Fig. 2a–e). In 11 tissues (from 4 autopsies and 3 biopsies), subpial demyelination was found within the cortex. Although perivascular complement deposition was almost absent at the demyelinating lesions within the cortex, 64% (7/11) of tissues showed C4d deposition on myelin sheaths or their debris in the subpial area or at the demyelinating border (Fig. 2m–r).

##### Deposition of different complement components within the lesions

**Fig. 3:**
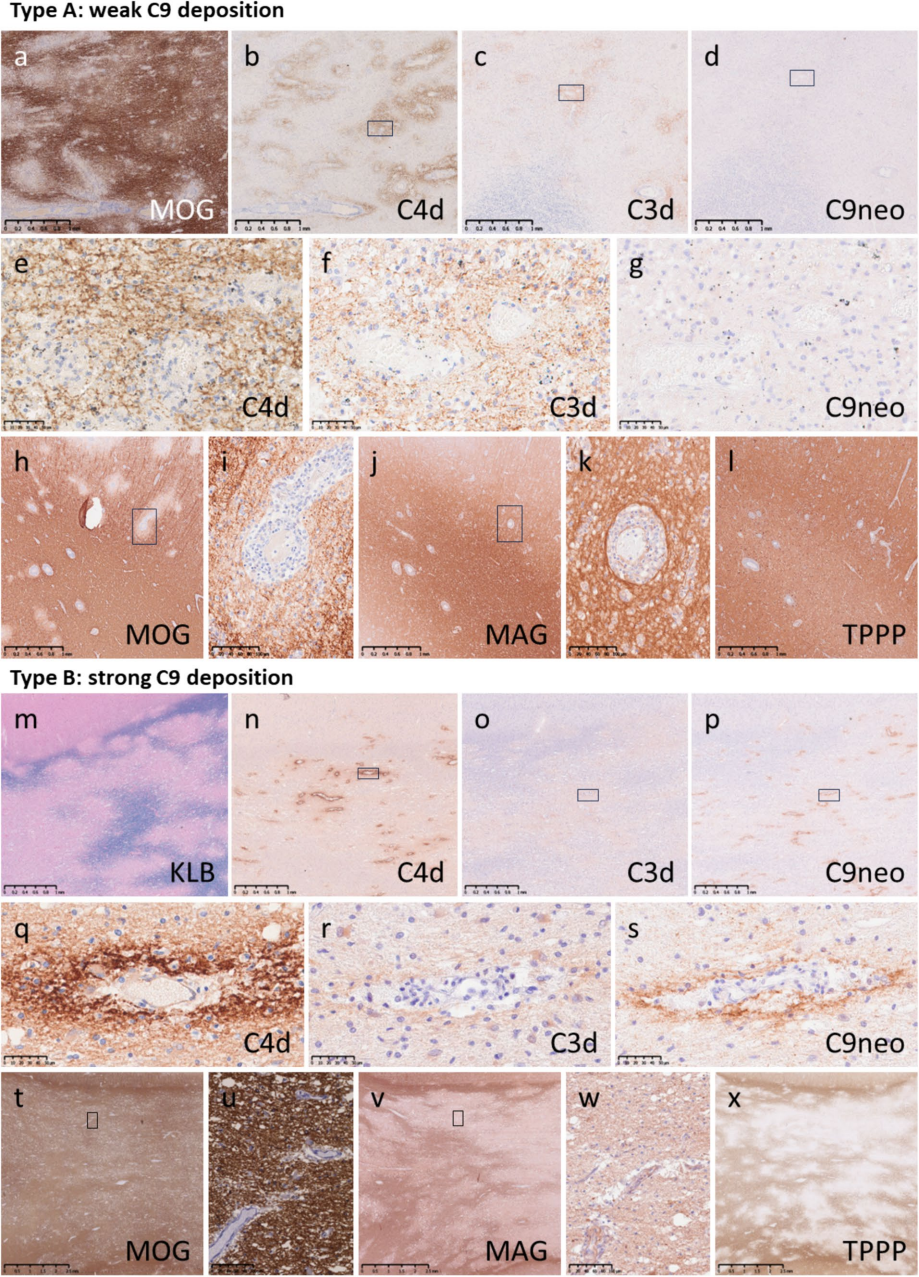
Two different complement deposition patterns in MOGAD. Type A lesions in MOGAD. a–g Cerebellum (MOG-a4). Numerous perivenous demyelinating lesions were observed (a, MOG). C4d (b and e) and C3d (c and f) were clearly deposited around perivenous demyelinating lesions, whereas C9neo (d and g) was very weakly deposited or nearly absent. h–l Brain (MOG-a6). In type A lesions, the degree of MOG staining (h and i, MOG) was severely diminished relative to MAG staining (j and k, MAG), and TPPP-positive oligodendrocytes were well preserved (l, TPPP). i and k Magnified images of the boxed areas in panels h and j, respectively. Type B lesions in MOGAD. m–x Brain (MOG-a2). Numerous perivenous demyelinating lesions were observed (m, KLB). Consistent with perivenous demyelinating lesions, C4d (q), C3d (r) and C9neo (s) were deposited in perivascular regions. In type B demyelinating lesions, MAG staining (v and w, MAG) was severely diminished relative to MOG staining (t and u), and TPPP-positive oligodendrocytes were severely damaged and depleted (x, TPPP). u and w Magnified images of the boxed areas in panels t and v. Scale bars: a–d, h, j, m–p, t and v = 1.0 mm; e–g and q–s = 50 µm; i, k, u and w = 100 µm. *KLB* Klüver-Barrera, *MAG* myelin-associated glycoprotein, *MOG* myelin oligodendrocyte glycoprotein, *MOGAD* myelin oligodendrocyte glycoprotein antibody-associated disease, *TPPP* tubulin polymerization-promoting protein

The staining pattern of C3d was generally similar, but the staining intensity of C3d was lower than that of C4d. Regarding C9neo, two different staining patterns were observed in PVD. In some lesions, C9neo staining was considerably weaker than C4d, or C9neo staining was nearly absent (type A lesions) (Fig. 3a–g), whereas marked C9neo deposition was detected in other lesions (type B lesions) (Fig. 3m–s). In type A lesions, oligodendrocytes were relatively well preserved within the area of demyelination, some of which showed more intense loss of MOG staining than of MAG staining (Fig. 3h–l). However, in type B lesions, oligodendrocytes were severely depleted, and the loss of MAG suggested destruction of their distal processes (Fig. 3t–x). Most biopsy samples showed type A lesions (8 with type A lesions vs. 1 with type B lesions), whereas 50% of the autopsy samples showed type B lesions (3 with type A lesions vs. 3 with type B lesions). The only biopsy sample that showed type B lesions was derived from the same patient that showed type B lesions at autopsy; these samples were taken at different times. Patients with type B lesions were significantly older than those with type A lesions (mean age 66.7 vs. 32.5 years; *p* = 0.025). Clinically, Type B lesions were associated with higher mortality and poorer treatment responsiveness, with fatal outcomes in all Type B cases (3/3) and no favorable response to corticosteroid therapy, in contrast to Type A lesions (4/11 fatalities; 6/9 steroid-responsive cases). Among the tissue with confluent demyelinating lesions, C9neo deposition was observed at the edge of the demyelinating lesions in 6.7% of samples (1/15) (Supplemental Fig. 2f–i), on the myelin sheath inside the demyelinating lesions in 26.7% of samples (4/15), and around vessels inside the demyelinating lesions in 6.7% of samples (1/15) (Supplemental Fig. 2j−o). C9neo deposition was completely absent in areas of subpial demyelination.

##### Differences in perivascular complement deposition between NMOSD and MOGAD patients

**Fig. 4:**
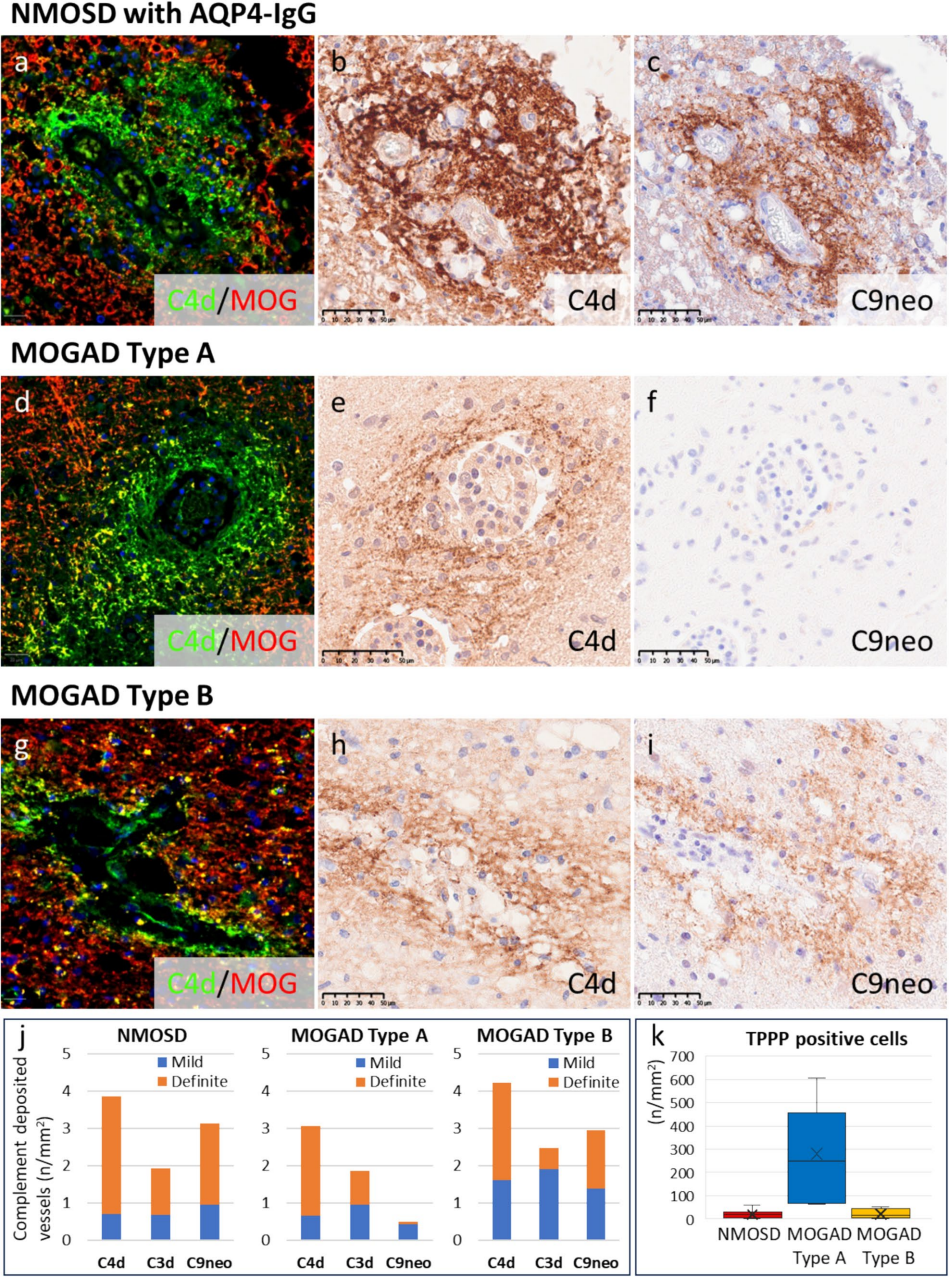
Differences between complement deposition patterns in NMOSD and MOGAD. **a–c** NMOSD with AQP4-IgG (NMO-a1). Complement deposition in the rosette pattern. C4d (**a**, green) did not colocalize with MOG (a, red) (**a**). The staining of C4d (b) and C9neo (**c**) was quite similar. d–f Type A MOGAD lesions (MOG-a3). C4d deposition (**d**, green) around perivenous demyelinating lesions expressing MOG (**d**, red). Compared with the deposition of C4d (**d**), the deposition of C9neo was very weak (f). g–i Type B MOGAD lesions (MOG-a2). C4d deposition (g, green) around perivenous demyelinating lesions expressing MOG (**g**, red), as observed in type A lesions. The deposition of C4d (**h**) and C9neo (**i**) was observed at the same level. **j** There was no significant difference in the number of vessels with C4d deposition per unit area between MOGAD patients and NMOSD patients, whereas the number of C9neo-deposited vessels per unit area was significantly lower in type A MOGAD lesions than in type B MOGAD or NMOSD lesions. **k** The number of TPPP-positive oligodendrocytes was preserved in type A MOGAD but markedly reduced in type B MOGAD and NMOSD lesions. Scale bars: a, d and g = 20 µm; b–c, e–f and h–i = 50 µm. *MOG* myelin oligodendrocyte glycoprotein, *MOGAD* myelin oligodendrocyte glycoprotein antibody-associated disease, *NMOSD* neuromyelitis optica spectrum disorder

Although similar patterns of complement deposition are observed in NMOSD and MOGAD, NMOSD is characterized by the constant deposition of complement in contact with the astrocytic glia limitans, and MOGAD is characterized by complement deposition on myelin (Fig. 4a–c). In MOGAD, both type A and type B lesions had similar staining patterns for C4d, with C4d colocalizing with perivascular myelin sheaths (Fig. 4d–e and g–h); however, as mentioned above, type A lesions had limited C9neo deposition, whereas type B lesions showed strong staining for C9neo (Fig. 4f and i). Whereas perivascular C4d deposition was frequently observed in all type A and type B lesions in MOGAD patients and NMOSD patients (NMOSD vs. MOGAD type A vs. MOGAD type B: 3.9 ± 1.9 vs. 3.1 ± 0.6 vs. 4.2 ± 2.5/mm^2^), C9neo deposition was much lower in type A lesions (NMOSD vs. MOGAD type A vs. MOGAD type B: 3.1 ± 1.0 vs. 0.48 ± 0.7 vs. 2.9 ± 3.5/mm^2^) (Fig. 4j), and the ratio of C9neo-positive vessels to C4d-positive vessels was very low in type A lesions (NMOSD vs. MOGAD type A vs. MOGAD type B: C3d/C4d ratio, 49.5% vs. 60.7% vs. 53.6%; C9neo/C4d ratio, 81.0% vs. 15.8% vs. 69.6%). TPPP-positive oligodendrocytes were well preserved in MOGAD type A lesions but severely depleted and lost in type B lesions, which was comparable to what was observed in NMOSD lesions (NMOSD vs. MOGAD type A vs. MOGAD type B: 19.6 ± 15.3 vs. 280.3 ± 201.1 vs. 22.3 ± 22.5/mm^2^) (Fig. 4k).

#### MS

##### Clinical characteristics and treatment status of the MS cohort

Information on disease course was available for all 18 patients included in the study. However, clinical information was incomplete in 5 patients, and detailed treatment data were available for only 11 patients. The median disease duration was 276 months (range, 16–504 months), and the median interval between the last clinical relapse and biopsy or autopsy was 138 months (range, 5–216 months). With respect to disease course, none of the patients exhibited a monophasic course; 5 patients (28%) had a relapsing course, while the majority, 13 patients (72%), showed a progressive course. At the time of autopsy, all patients (18/18, 100%) were classified as being in the chronic phase. Regarding treatment status, MS disease-modifying therapies were administered in 3 of the 11 patients with available treatment data, including fingolimod in 2 patients and interferon-β in 1 patient, while conventional immunosuppressive therapy with azathioprine was used in 2 patients.

##### Patterns of complement deposition in typical MS

**Fig. 5:**
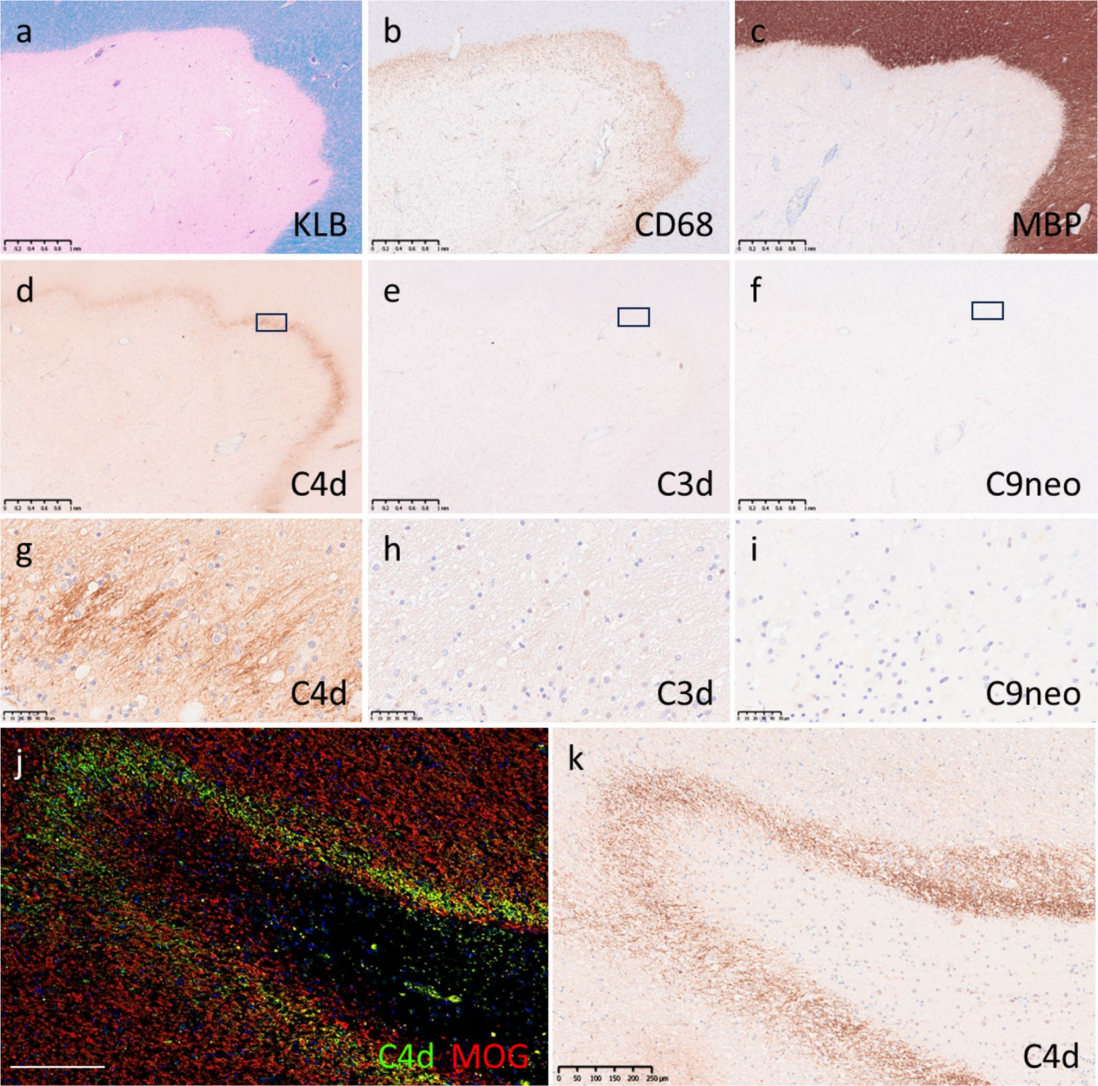
Complement deposition patterns in MS. a–i Brain (MS-15). Typical confluent demyelinating lesions in multiple sclerosis (**a**, KLB). Macrophages accumulated at the margins of demyelinating lesions, showing characteristic mixed active/inactive lesions (**b**, CD68). The borders of demyelinating lesions were quite distinct (**c**, MBP). C4d was deposited surrounding demyelinating lesions (d and g, C4d). Weak deposition was also observed for C3d (c and h, C3d) but not for C9neo (**f** and **i**, C9neo). **g–h** Magnified images of the boxed areas in panels **d–f**. **j–k** Brain (MS-7). C4d (j [green], k [brown]) was localized on myelin sheaths (**j** [red], MOG) near demyelinating lesions. Scale bars: a–f = 1.0 mm; g–i = 50 µm; **j–k** = 250 µm. *KLB* Klüver-Barrera, *MBP* myelin basic protein, *MOG* myelin oligodendrocyte glycoprotein, *MS* multiple sclerosis

Typical MS lesions were found in 18 patients in 47 tissue blocks (29 brain, 11 brainstem, 4 spinal cord and 3 optic nerve tissue blocks) (Table 1). First, we consistently identified complement deposition in mixed active/inactive lesions (MALs) (Fig. 5a–c), which are a hallmark of MS. We detected C4d deposition around MALs (Fig. 5d and g); similar findings were observed for C3d, although the staining intensity was much weaker (Fig. 5e and h). C9neo deposition was not observed around any of the MALs (Fig. 5f and i). C4d was mostly deposited on morphologically intact myelin sheaths in the periplaque white matter around the demyelinating lesions (Fig. 5j–k). Close to the edges of the demyelinating lesions, C4d was sometimes deposited on degenerating myelin sheaths and oligodendrocytes (Supplemental Fig. 3a−l). Complement deposition was scarce within the demyelinating lesions, but oligodendrocyte-like staining (Supplemental Fig. 3g and k) and myelin debris-like staining (Supplemental Fig. 3h and i) were occasionally present. Complement was also observed inside macrophage granules, predominantly within the lesions or in the perivascular space (Supplemental Fig. 3m−o). The deposition of each perivascular complement was detected in MS (Supplemental 3p−r), but unlike in MOGAD or NMOSD, complement deposition was detected in normal-appearing white matter (NAWM) near demyelinating lesions. These findings were specific for C4d/C3d and were not found for C9neo (Supplemental Fig. 3p-r).

##### Relationship between the perilesional pattern of complement and stage of demyelination or disease duration in MS patients

**Fig. 6:**
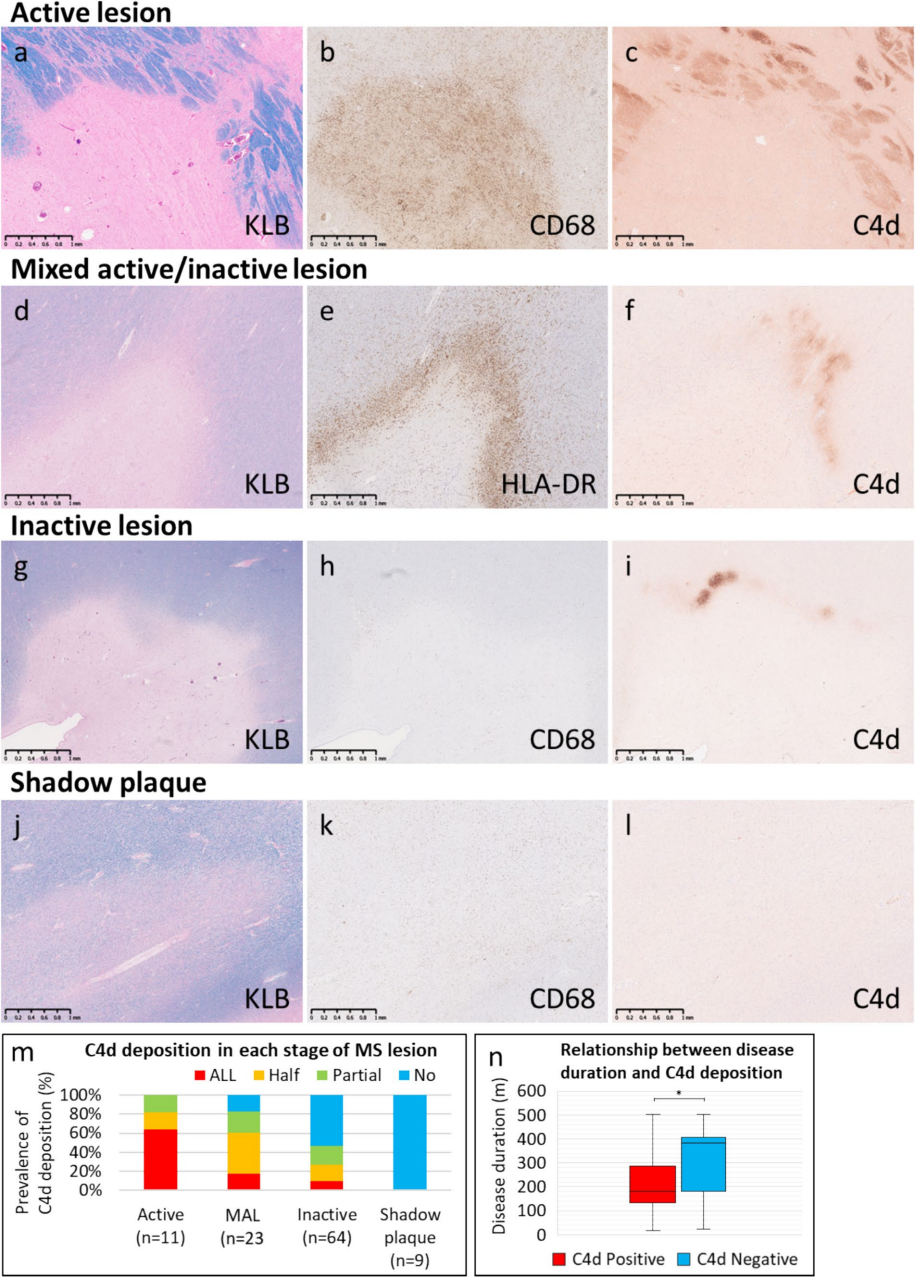
Relationship between complement deposition and lesion stage in MS patients. **a–c** Active lesion. Confluent demyelinating lesions (a, Klüver-Barrera) were covered with numerous CD68-positive macrophages (**b**). C4d was extensively deposited on the myelin sheath around demyelinating lesions (c). d–f Mixed active/inactive lesion. HLA-DR-positive macrophages and microglia (**e**) accumulated at the margins of the confluent demyelinating lesion (**d**, Klüver-Barrera). C4d was deposited on some myelin sheaths in the peri-plaque white matter around the demyelinating lesions (**f**). **g–i** Inactive lesion. Extensive demyelinating lesions were present (**g**, Klüver-Barrera), but macrophages within demyelinating lesions were scant (**h**, CD68). C4d was deposited in a portion of the myelin sheath surrounding demyelinated lesions (**i**). j–l Shadow plaques. Oval-shaped remyelinating lesions composed of thin myelin sheaths (**j**, Klüver-Barrera). Macrophages were nearly completely absent (**k**, CD68). No C4d deposition was observed (**l**). **m** Percentage of C4d deposition around demyelinating lesions in each stage of multiple sclerosis. The more active the demyelination was, the more frequent and extensive the complement deposition was. **n** Relationship between disease duration and complement deposition. Lesions with complement deposition had a shorter interval between onset and death (**n**). * p < 0.01. Scale bar: a–l = 1 mm

We evaluated the association between complement deposition of the perilesional pattern and the stage of demyelination (Fig. 6m). In active lesions, C4d deposition was not only localized to the lesion margins but also extensively deposited on the myelin sheath around the lesions (Fig. 6a–c). Conversely, in MALs, C4d was localized at the rims of demyelination, showing continuous (Fig. 5a–d) or discontinuous/partial deposition (Fig. 6d–f). In inactive lesions, complement deposition was less frequent overall (Fig. 6m), but the qualitative findings were similar to those for MALs (Fig. 6g–i); however, in inactive lesions with remyelination, complement deposition was consistently absent (Fig. 6j–l and m).

Patients with complement deposition had a significantly shorter disease duration than those without complement deposition did (interval between onset and death [mean ± SD, months]: with complement vs. without complement = 197.2 ± 144.5 vs. 305.5 ± 136.6, *p* < 0.01) (Fig. 6n).

##### Patterns of complement deposition in control tissues

We evaluated complement deposition patterns in autopsy tissues from patients with stroke, bacterial CNS infections, glioblastoma and primary malignant CNS B-cell lymphoma. The patterns of complement deposition clearly differed between the acute and chronic phases of cerebral infarction. In acute ischaemic infarct lesions showing oedema, tissue pallor, red neurons and abundant apoptotic nuclei, perivascular C3d and C4d deposition was invariably prevalent (4/4), whereas marked C9neo deposition was not observed. Similar perivascular deposition of C3d and C4d was observed in subacute ischaemic infarct tissues with loss of astrocytes and vessel sprouting (4/4). One patient showed perivascular dot-like C9neo deposition (Supplemental Fig. 4). Conversely, in residual lesions in the chronic phase of cerebral infarction featuring glial scar formation, mild complement reactivity was observed in macrophages within the lesion.

In three patients with bacterial meningoencephalitis, complement deposition was observed on the meninges and accompanied by inflammatory cell infiltration. In one patient with sepsis, a fibrous pattern of complement deposition around veins was observed (Supplemental Fig. 5).

In all the patients with glioblastoma (5/5) and 3 out of the 4 patients with malignant B-cell lymphoma, strong deposition of C4d, C3d, and C9neo in the neuropil and cellular components was observed in necrotic tissue, but no perivascular pattern was observed in vital brain or tumour tissue.

## Discussion

In the present study, we identified distinctive patterns of complement deposition in NMOSD, MOGAD, and MS (the findings are summarized in Table 2). In NMOSD, complement deposition in a rosette pattern and/or a rim pattern was observed around blood vessels inside astrocyte-destructive lesions, as previously reported [22, 39]. The pattern of complement deposition in NMOSD was uniform in each stage of the disease. In particular, in the acute stage, complement deposition was found in almost all tissues, and the C4d, C3d and C9neo staining patterns were similar. These findings likely reflect the finalization of the complement cascade in the MAC, supporting previous data, which implicated complement in the pathogenicity and reported high efficacy of complement inhibitors in NMOSD [34, 35]. However, careful attention should be given to the fact that C9neo deposition is markedly reduced in the subacute phase and is not observed in the chronic phase when histology is used for diagnosis [39]. We previously reported that in NMOSD, C3d deposition is observed in a rim pattern through the chronic phase [39], but in this study, we found C4d deposition to be similar to that of C3d. These findings suggest that complement activation occurs continuously at the foot processes of astrocytes forming the perivascular glia limitans, but is regulated in the early stages of MAC formation. While persistent activation of the classical pathway — most likely mediated by the AQP4 antibodies — is a plausible candidate mechanism, evidence of C4 activation may also reflect involvement of the lectin pathway in NMOSD pathogenesis. Although the mechanisms remain incompletely understood, these findings related to NMOSD relapse warrant further investigation.

**Table 2:** Summary of complement deposition patterns in NMOSD, MOGAD and MS

Disease	NMOSD	MOGAD		MS
		Type A	Type B	
Primary target	Astrocytes	Myelin > Oligodendrocytes	Myelin and Oligodendrocytes	Myelin and Oligodendrocytes
Demyelination pattern	Extensive demyelination associated with astrocyte destruction and distal oligodendrogliopathy	Perivenous demyelination and its fused lesions, subpial demyelination		Confluent demyelination, intra cortical and subpial demyelination
Astrocyte reaction	Loss in acute lesions, fibrous gliosis with honeycomb appearance in chronic lesions	Reactive in acute lesions		Reactive in acute lesions, gliosis in chronic lesions
Oligodendrocyte reaction	Loss	Relatively preserved	Loss	Relatively preserved
Complement				
Deposition pattern	Vasculocentric (rosette/rim pattern), foot and degenerated cell body of astrocytes	On myelin sheaths at the border of demyelinating lesions		On myelin sheaths at peri-plaque white matter adjacent to demyelinating lesions, degenerated oligodendrocytes
Acute lesions				
C4d	+ ~ + + +	+ + ~ + + +	+ + ~ + + +	+ ~ + +
C3d	+ ~ + + +	+ + ~ + + +	+ ~ + +	± ~ +
C9neo	+ ~ + + +	- ~ +	+ + ~ + + +	-
Subacute or chronic active lesions				
C4d	+ ~ + + +	+ ~ + +	+ ~ + + +	- ~ + +
C3d	+ ~ + + +	+ ~ + +	+ ~ + +	- ~ +
C9neo	- ~ +	- ~ ±	+ ~ + +	-
Chronic inactive lesions				
C4d	+ ~ + + + (only rim pattern)	-	NA	- ~ +
C3d	+ ~ + + + (only rim pattern)	-	NA	- ~ +
C9neo	-	-	NA	-

-: none, + : mild, +  + : moderate, +  +  + : severe

NA: Not Applicable

In MOGAD, ADEM-like perivenous demyelination was observed from the corticomedullary junction to the white matter in the acute phase, as has been clearly demonstrated in previous studies [11, 37]. We detected C4d deposition in approximately 90% of such perivenous demyelinating lesions. In addition, C4d colocalized with myelin, likely due to activation of the initial steps of the classical pathway by MOG-Ab. These findings suggest that perivascular myelin sheath disruption involves complement in the acute lesions of MOGAD. However, histopathological analysis of MOGAD samples revealed two distinct groups in terms of C9neo deposition: type A lesions showed limited deposition of C9neo even in the acute phase, whereas type B lesions showed marked deposition of C9neo. Furthermore, markers of oligodendroglial injury differed between type A and type B lesions. In type A lesions, oligodendrocytes were relatively preserved, whereas in type B lesions, oligodendrocytes were markedly depleted. In our study, 73% of MOGAD patients presented with weak or absent C9neo deposition (Type A). Our findings are in line with previous findings, as MOG-Ab can induce complement-dependent cytotoxicity (CDC) [15, 31], but the cytotoxicity of MOG-Ab is known to be lower than that of AQP4-Ab [20]. Hence, our finding of less frequent C9neo deposition in MOGAD patients than in NMOSD patients could reflect lower CDC activity and could explain, in part, why MOGAD has a better prognosis than NMOSD [7]. Our data are also compatible with the recently reported findings regarding complement levels in the serum and CSF. Cho et al. comprehensively examined complement-related protein levels in the serum of patients with MOGAD and NMOSD and reported that the levels of C1 inhibitors, factor H, and iC3b were higher in samples from patients with MOGAD during the attack phase but that sC5b-9 levels were elevated only in patients with NMOSD [6]. In addition, Kaneko et al. [14] and Villacieros-Álvarez et al. [40] compared the levels of complement components in NMOSD, MOGAD, and MS and reported that C3a and C5a levels in the CSF [14] and serum [40] were markedly elevated in both NMOSD and MOGAD compared with MS, whereas CSF C5b-9 levels were elevated only in NMOSD [14]. However, both reports also revealed that there was a subgroup of patients with MOGAD with high levels of C5b-9 comparable to those in patients with NMOSD, and the disability-related outcomes in these patients were worse [14, 40]. These findings are consistent with our observation that type B lesions in MOGAD were associated with a more severe clinical course, including poor responsiveness to corticosteroids and fatal clinical outcomes. Moreover, patients with type B lesions were significantly older than those with type A lesions, raising the possibility that age-related factors, such as alterations in CNS tissue vulnerability or complement regulation, may promote MAC formation. Taken together, the complement system appears to be generally activated in MOGAD, but interindividual differences in MAC formation, which are influenced by both patient-related factors and disease-specific immune mechanisms, may decisively determine the pathological heterogeneity and clinical severity in MOGAD.

What causes the difference in complement activity in MOGAD, particularly MAC formation? MOG-Abs are thought to be composed mainly of IgG1 [42], but a recent analysis revealed that there are cases in which the IgG3 subclass, which has higher complement activity, is dominant [13]. Therefore, whether the difference in IgG subclass affects the formation of MACs will be addressed in future research. Another possibility is the influence of coexisting autoantibodies. MOGAD can be accompanied by other autoantibodies, particularly NMDA receptor antibodies [10, 17]. In addition, conformational antibodies against proteolipid protein-1 (PLP1) were identified in 8% (7/89) of patients with MOGAD, and the severity of the disease was greater in patients with PLP1-IgG [24]. In this study, owing to the lack of sufficient residual serum, it was impossible to perform a comprehensive evaluation of autoantibodies; however, future accumulation of samples for this purpose is warranted. We also need to consider the interaction of MOG-specific T/B cells with MOG-Ab. The role of lymphocytes in MOGAD is not fully understood, but we have accumulated a wide range of information on experimental autoimmune encephalomyelitis against MOG (MOG EAE). MOG EAE can be induced in several rodent species with either various peptide fragments or the full MOG protein [27]. Therefore, different models of EAE differ in their dependency on B-cell-dependent antigen presentation, with EAE induced by full-length MOG being dependent on B cells, which is not the case for EAE induced by MOG peptide fragments [23]. The dependence of pathogenicity on antibodies as well as their ability to fix the complement system and form MACs seems to vary between the many EAE models, which differ based on species and the various combinations of T cell, B-cell and antibody specificities. As a common denominator among most MOG EAE models, however, demyelination and clinical disease seem to depend on pathogenic MOG-autoreactive T cells (which are also needed to open the blood–brain barrier to allow the entry of pathogenic autoantibodies), whereas the presence of MOG-Ab may also increase demyelination and clinical severity [18]. For example, in an EAE model induced in Lewis rats with MBP-specific T cells and MOG antibodies, T cells induce brain inflammation and BBB opening, whereas the actual demyelinating potential of the model is linked to the antibodies and their ability to fix the complement system and form MACs, suggesting CDC [32]. In another study on different variants of EAE using MBP-specific T cells and MOG-Ab, liposome-mediated macrophage depletion significantly reduced pathogenicity, possibly suggesting antibody-dependent cellular phagocytosis (ADCP) [12]. Therefore, MOG-Ab in EAE seems to participate in tissue destruction through various pathogenic pathways, possibly showing some similarities to MOGAD.

In contrast, the complement deposition pattern in MS patients differed from those observed in NMOSD and MOGAD patients and was characterized by the deposition of C4d, an early complement activation marker, along the myelin sheaths at the margins of demyelinating lesions. Although the extent and frequency of complement deposition varied depending on the lesion stage, the basic characteristics were similar, and they were observed uniformly in MS patients who presented with typical confluent demyelination. Interestingly, patients with complement deposition had a shorter disease duration than those without complement deposition did. Furthermore, because complement deposition did not occur in the lesions where the myelin sheaths had regenerated, it was speculated that complement components act as factors in the expansion and progression of demyelinating lesions and potentially in the inhibition of remyelination.

Our patients with typical MS pathology did not display pattern 2 lesions, which are characterized by C9neo deposition within active confluent demyelinating lesions [21]. The discrepancies in the presence of complement components in MS lesions reported by Lucchinetti et al. [21] could be at least in part explained by the older ages of our patients. In our study, active lesions occurred mainly in the postdemyelinating stage in patients who were unlikely to have highly fulminant early active lesions (macrophages with MAG + or MOG + degradation products), which are required to detect C9neo deposition in MS [21]. In contrast, our findings of frequent C3d and C4d deposition are in line with those for a previously published cohort with comparable demographic characteristics [3]. Moreover, these findings are consistent with the observations reported by Cooze et al., who demonstrated predominant upstream complement activation, including early complement components, with minimal terminal complement complex formation in chronic progressive MS lesions [8]. These pathological observations are also in good agreement with in vivo imaging studies by Dal-Bianco et al., who demonstrated chronic active MS lesions characterized by persistent inflammatory activity at lesion rims, which are thought to drive slow lesion expansion and neurodegeneration [9]. Absinta et al. furthermore implicated complement C1q as another early complement factor and determinant of lesion expansion-promoting myeloid cell activity at chronic active lesion rims [1]. Thus, we confirm that upstream complement activation frequently occurs even in the progressive stages of MS and that downstream MAC activation seems to occur very rarely or not at all in typical and longstanding MS. This pattern of predominant upstream complement activation without terminal MAC formation provides a pathological basis for recent studies linking complement activation products in CSF, in particuluar C4a, to more severe disease progression in MS and prevalence of paramagnetic rim lesions as the MRI equivalent of chronic active lesions [29, 30]. Moreover, we suggest C4d as a more robust complement screening marker than C3d and C9neo in MS, as its perilesional rim-associated deposition might aid in the diagnosis of active/mixed active MS lesions and even a subgroup of inactive MS lesions. In this context, Watkins et al. reported C9neo deposition in cortical gray matter lesions using frozen tissue sections, indicating that complement activation can proceed to the terminal stage in specific anatomical compartments and pathological contexts [43]. Therefore, differences in complement deposition patterns among studies likely reflect lesion location, disease stage, and methodological approaches.

As described above, we have shown that there are characteristic complement deposition patterns in NMOSD, MOGAD and MS. These findings are considered highly useful for the differential diagnosis of each types of inflammatory demyelinating lesion. However, nonspecific complement deposition was also observed in the control group, particularly in conditions involving infection (acute phase meningitis), and in lesions featuring tissue necrosis, such as acute cerebral infarction, lymphoma and glioblastoma samples. Because some of these cases showed complement deposition around blood vessels, it is essential to properly evaluate them, including for factors other than complement deposition, such as the characteristics of demyelinating lesions and the presence or absence of astrocytopathy, including AQP4 loss. Careful consideration is also needed when biopsy specimens are being examined, as they may also contain nonspecific artefacts.

The differences in complement deposition patterns among patients with different IDDs could also be useful for understanding disease pathogenesis and developing appropriate therapies. We found that in all the diseases included in our present study, complement components were deposited at the site of active tissue damage. Complement activation may be induced by the binding of antibodies to their target structure, which leads to activation of the entire complement cascade and the formation of MACs. When activation is not fully sufficient or is partially blocked by complement inhibitor proteins, MAC formation may be inhibited. However, even in such cases, complement components may augment antibody-dependent cellular cytotoxicity by promoting the phagocytosis of autoantibody-coated targets. In addition, certain complement components are produced by neurons and glia in response to tissue injury. They can then accumulate on damaged tissue, being recognized by complement receptors on activated macrophages and microglia and promoting the clearance of tissue debris from lesions. While in the first scenario, blocking the formation of MACs is a promising therapeutic option, in the second scenario, upstream complement components must be inhibited. In the third scenario, complement inhibition is likely detrimental, as clearance of debris is a prerequisite for neural plasticity and remyelination.

In this study, we identified the characteristic complement deposition patterns for each disease. In summary, complement deposition was observed on astrocytes in NMOSD, whereas it was present on myelin sheaths in MOGAD and MS, reflecting differences in the primary targets of the diseases. Complement deposition occurred at the glia limitans from the acute phase to the chronic phase in NMOSD, accompanied by extensive MAC formation in the acute phase. These findings suggest that NMOSD pathophysiology involves astrocyte destruction associated with CDC specifically targeted to AQP4, which is densely expressed in astrocyte foot processes at the glia limitans. Conversely, MOGAD patients showed frequent complement deposition at the lesion borders of perivenous demyelination, where early complement split products bound to the myelin sheaths surrounding the demyelinating lesions and moved away from the perivascular area with expanding demyelination, distinct from what was observed in NMOSD. MAC formation was generally lower in MOGAD than in NMOSD, but C9neo deposition was strong in some cases, suggesting heterogeneity in complement activity, particularly regarding MAC formation. In typical MS, early complement split product deposition was observed on the myelin sheath at the periphery of confluent demyelinating lesions. However, the absence of MAC formation and the presence of C4d deposition around even some chronic inactive MS lesions were features that differed from those of MOGAD, suggesting the possible involvement of early complement split products in slowly progressive tissue injury in chronic MS. Although differences in complement deposition patterns between different IDDs is considered quite useful in differentiating the pathology of the diseases, the significance of these differences remains unknown. Additionally, this study has several limitations. Detailed clinical information was unavailable for some cases; notably, the serum status of AQP4-Ab and MOG-Ab was unknown for many MS cases, and therefore the possibility of disease misclassification cannot be entirely excluded. Immunotherapy had been administered in a relatively large proportion of cases, which may have influenced the histopathological findings. Furthermore, the availability of pathological specimens was uneven across disease stages, with limited representation of acute-phase MS lesions and chronic-stage MOGAD lesions, which may have affected the interpretation of complement activation patterns. In addition, immunohistochemical analyses were performed on formalin-fixed paraffin-embedded tissue, which is associated with reduced sensitivity for certain complement components and may underestimate the extent of complement activation. Future studies incorporating well-characterized clinical data, a broader range of disease stages, and complementary methodological approaches will be essential not only to overcome these limitations but also to further elucidate the role of complement activation in the pathophysiology of inflammatory demyelinating diseases.

## Fundings

This study was supported in part by KAKENHI (#23K06959, #24K10634) from the Ministry of Education, Culture, Sports, Science and Technology (MEXT), the Grants-in-Aid for Scientific Research from the Ministry of Health, Labour and Welfare of Japan, and the National Institute of Neurological Disorders and Stroke of the National Institutes of Health under Award Number R01NS114227, the Austrian Science Fund (FWF grant 10.55776/PAT6054424 to MB and FWF grant I6565-B to RH), and the Austrian Research Promotion Agency (FFG), project number FO999920011 to RH.

## Supplementary Information

Below is the link to the electronic supplementary material.

Supplementary file1 (DOCX 14515 KB)

## Abbreviations

Ab: Antibody
ADCP: Antibody-dependent cellular phagocytosis
ADEM: Acute disseminated encephalomyelitis
AP: Alkaline phosphatase
AQP4: Aquaporin-4
CBA: Cell-based assays
CDC: Complement dependent cytotoxicity
CNS: Central nervous system
DAB: Diaminobenzidine hydrochloride
DMT: Disease modifying therapy
EAE: Experimental autoimmune encephalomyelitis
GFAP: Glial fibrillary acidic protein
HE: Hematoxylin and Eosin
HRP: Horseradish peroxidase
IDD: Inflammatory demyelinating diseases
IVIg: Intravenous immunoglobulin
IVMP: Intravenous methylprednisolone
KLB: Klüver–Barrera
MAC: Membrane attack complex
MAG: Myelin associated glycoprotein
MBP: Myelin basic protein
MOG: Myelin oligodendrocyte glycoprotein
MOGAD: MOG antibody-associated disease
MS: Multiple sclerosis
NMDA: N-methyl-D-aspartic acid
NMOSD: Neuromyelitis optica spectrum disorder
PBS: Phosphate buffered saline
PE: Plasma exchange
PLP1: Proteolipid protein-1
PVD: Perivenous demyelination
RT: Room temperature
SEL: Slowly expanding lesion
TBS: Tris-buffered saline
TPPP: Tubulin polymerization-promoting protein
