# Supplementary material for: Characteristic patterns of complement deposition in NMOSD, MOGAD, and MS
Source: Acta Neuropathol. 2026 Feb 9;151(1):13. doi: 10.1007/s00401-026-02985-9 (PMC12886220; doi:10.1007/s00401-026-02985-9)
Supplement: Supplementary file 1 — Supplementary file1 (DOCX 14515 KB) [file 401_2026_2985_MOESM1_ESM.docx]

**Supplementary data**

Manuscript for Acta Neuropathologica (Original Article)

**Title:**

**Characteristic patterns of complement deposition in NMOSD, MOGAD, and MS**

**Authors**

*Yoshiki Takai, MD, PhD^1,2^; *Simon Hametner, MD, PhD^3^; Christian Riedl, MD^3^; Tatsuro Misu, MD, PhD^1^; Toshiyuki Takahashi, MD, PhD^1,4^; Hiroyoshi Suzuki, MD, PhD^5^; Norio Chihara, MD, PhD^6^; Masashi Watanabe, MD^7^, Hiroaki Miyahara, MD; PhD^8^; Mari Yoshida, MD, PhD^8^; Yasushi Iwasaki, MD, PhD^8^; Takashi Suzuki, MD, PhD^2^; Franziska Di Pauli, MD, PhD^9^; Stephan Bramow, MD, PhD^10^; Guy Laureys, MD, PhD^11^; Brenda Banwell, MD^12^; Sara Mariotto, MD, PhD^13^; Kazuo Fujihara, MD, PhD^1,14^; Masashi Aoki, MD, PhD^1^; Monika Bradl, PhD^15^; Hans Lassmann, MD, PhD^15^; Romana Höftberger, MD^3^.

^*^ These two authors contributed equally to this work

**Corresponding author**

Yoshiki Takai

Department of Neurology, Tohoku University Graduate School of Medicine, Sendai 980-8574, Japan

Tel +81-22-717-7189

Fax +81-22-717-7192

E-mail: yoshiki.takai.e6@tohoku.ac.jp

**Supplementary Table 1: Summary of clinical presentations and pathological findings in inflammatory demyelinating disease**

| Pt.No | Age | Sex | Disease duration (m) | Interval duration (m)* | Clinical diagnosis | pathological characteristics | Stage of the lesions** |
| --- | --- | --- | --- | --- | --- | --- | --- |
| NMO-a1 | 78 | F | 2 | 0.4 | NMOSD+ | Astrocytopathy | Acute～Subacute |
| NMO-a2 | 63 | M | 0.6 | 0.6 | NMOSD+ | Astrocytopathy | Subacute |
| NMO-a3 | 57 | M | 8 | 0.5 | NMOSD+ | Astrocytopathy | Subacute |
| NMO-a4 | 53 | F | 3 | 2 | NMOSD+ | Astrocytopathy | Subacute |
| NMO-a5 | 46 | F | 252 | 12 | NMOSD+ | Astrocytopathy | Chronic |
| NMO-a6 | 71 | F | 120 | 36 | NMOSD+ | Astrocytopathy | Chronic |
| NMO-a7 | 56 | F | 228 | 108 | NMOSD+ | Astrocytopathy | Chronic |
| NMO-a8 | 46 | F | 156 | 108 | NMOSD+ | Astrocytopathy | Chronic |
| NMO-a9 | 71 | F | 252 | 22 | NMOSD+ | Astrocytopathy | Chronic |
| NMO-a10 | 87 | M | 20 | 15 | NMOSD+ | Astrocytopathy | Chronic |
| NMO-a11 | 23 | F | 148 | 3 | NMOSD | Astrocytopathy | Acute~Chronic |
| NMO-a12 | 23 | F | 240 | 0.5 | NMOSD | Astrocytopathy | Acute~Chronic |
| NMO-a13 | 46 | F | 84 | 0.6 | NMOSD | Astrocytopathy | Subacute~Chronic |
| NMO-a14 | 72 | F | 5 | 5 | NMOSD | Astrocytopathy | Chronic |
| NMO-a15 | 22 | F | NA | NA | NMOSD+ | Astrocytopathy | Acute~Subacute |
| NMO-a16 | 78 | F | 2 | 2 | NMOSD+ | Astrocytopathy | Subacute~Chronic |
| NMO-a17 | 51 | F | 5 | 2 | NMOSD | Astrocytopathy | Acute~Chronic |
| NMO-a18 | 20 | F | 48 | 3 | NMOSD | Astrocytopathy | Acute~Chronic |
| NMO-b1 | 67 | F | 1.5 | 1.5 | NMOSD+ | Astrocytopathy | Subacute~Chronic |
| MOG-a1 | 31 | F | 5.5 | 5.5 | MOGAD | Confluent+Subpial DM | Acute~Subacute |
| MOG-a2 | 67 | F | 2.5 | 2.5 | MOGAD | PV+PV(fusion)+Subpial DM | Acute |
| MOG-a3 | 9 | M | 0.13 | 0.13 | MOGAD | PV+Subpial DM | Acute |
| MOG-a4 | 21 | F | 26 | 0.1 | MOGAD | PV+PV(fusion) + Subpial DM | Acute~Subacute |
| MOG-a5 | 9 | M | 0.25 | 0.25 | MOGAD | PV+Subpial DM | Acute |
| MOG-a6 | 52 | M | 0.75 | 0.75 | MOGAD | PV+PV(fusion)+Subpial DM | Acute |
| MOG-a7 | 81 | M | 0.4 | 0.4 | MOGAD | PV+PV(fusion) | Acute |
| MOG-b1 | 24 | F | 2 | 2 | MOGAD | PV+Subpial DM | Acute~Subacute |
| MOG-b2 | 16 | F | 96 | 96 | MOGAD | PV(fusion) | Chronic |
| MOG-b3 | 33 | M | 1 | 1 | MOGAD | PV | Acute |
| MOG-b4 | 64 | F | 0.5 | 0.5 | MOGAD | PV | Acute |
| MOG-b5 | 27 | M | 16 | 1 | MOGAD | Confluent+Subpial DM | Acute~Subacute |
| MOG-b6 | 47 | M | 2 | 2 | MOGAD | PV+PV(fusion) | Acute~Subacute |
| MOG-b7 | 62 | M | 32 | 0.75 | MOGAD | PV(fusion) | Acute~Subacute |
| MOG-b8 | 29 | F | 0.75 | 0.75 | MOGAD | Subpial DM | Acute |
| MOG-b9 | 58 | M | 2 | 2 | MOGAD | PV | Subacute |
| MOG-b10 | 32 | M | 0.75 | 0.75 | MOGAD | PV+PV(fusion) | Acute |
| MOG-b11 | 12 | F | 0.75 | 0.75 | MOGAD | Confluent+Subpial DM | Acute~Subacute |
| MOG-b12 | 28 | F | 10 | 1 | MOGAD | PV | Acute |
| MOG-b13 | 44 | F | 6 | 6 | MOGAD | Complete DM | Subacute~Chronic |
| MOG-b14 | 46 | M | 2 | 2 | MOGAD | No DM | Subacute~Chronic |
| MOG-b15 | 3 | F | 17 | <2 | MOGAD | PV+PV(fusion) | Acute~Subacute |
| MOG-b16 | 35 | M | 1 | 1 | MOGAD | Confluent | Acute |
| MOG-b17 | 53 | F | 168 | 2 | MOGAD | PV+PV(fusion) | Acute~Subacute |
| MOG-b18 | 4 | M | <1 | <1 | MOGAD | PV | Acute |
| MOG-b19 | 67 | F | 0.13 | 0.13 | MOGAD | PV | Acute |
| MOG-b20 | 57 | M | NA | NA | MOGAD | PV | Acute |
| MS-1 | 43 | M | 132 | 132 | PPMS | Confluent (MS type) | I |
| MS-2 | 60 | M | 180 | 78 | SPMS | Confluent (MS type) | I |
| MS-3 | 56 | M | 48 | 5 | RRMS | Confluent (MS type) | SEL, I |
| MS-4 | 34 | M | 24 | NA | SPMS | Confluent (MS type) | A, SEL |
| MS-5 | 46 | F | 216 | NA | RRMS | Confluent (MS type) | I |
| MS-6 | 59 | F | 444 | NA | RRMS | Confluent (MS type) | SEL, I |
| MS-7 | 44 | F | 252 | 216 | SPMS | Confluent (MS type) | PD, I |
| MS-8 | 55 | F | 16 | NA | RRMS | Confluent (MS type) | PD |
| MS-9 | 76 | F | NA | NA | PMS | Confluent (MS type) | I, Sh |
| MS-10 | 47 | F | 276 | 48 | SPMS | Confluent (MS type) | SEL, I |
| MS-11 | 69 | F | 504 | 168 | SPMS | Confluent (MS type) | I |
| MS-12 | 56 | F | 348 | 192 | SPMS | Confluent (MS type) | Sh |
| MS-13 | 61 | F | 396 | 180 | SPMS | Confluent (MS type) | PD, SEL, I, Sh |
| MS-14 | 52 | M | 288 | 108 | RRMS | Confluent (MS type) | SEL |
| MS-15 | 50 | F | 276 | 216 | SPMS | Confluent (MS type) | SEL, I |
| MS-16 | 60 | F | 408 | 12 | SPMS | Confluent (MS type) | SEL, I, Sh |
| MS-17 | 73 | M | 384 | 120 | SPMS | Confluent (MS type) | I |
| MS-18 | 68 | M | Incidental | Incidental | Incidental | Confluent (MS type) | SEL |

a = autopsy, b = biopsy in Pt.No. Age: at biopsy or at autopsy

*Interval duration = Interval between the last attack and biopsy/autopsy

**stage of multiple sclerosis: A=active lesion, PD=post-demyelinating lesion, SEL=slowly expanding lesion, I=inactive lesion, Sh=Shadow plaque.

DM: demyelination, F: female, M: male, MS: multiple sclerosis, NA: not applicable, NMO: neuromyelitis optica, NMOSD: NMO spectrum disorders with unknown serostatus, NMOSD+: NMOSD with aquaporin4 antibody, MOGAD: myelin oligodendrocyte glycoprotein antibody- associated disease, PPMS: primary progressive multiple sclerosis, PV: perivenous demyelination, RRMS: relapse remitting multiple sclerosis, SPMS: secondary progressive multiple sclerosis.

**Supplementary Table 2: Antibodies used for IHC**

| Target | Host | Cat Nr. | Protocol | Ag retrieval | Conc. | Secondary system | Company |
| --- | --- | --- | --- | --- | --- | --- | --- |
| Single staining | | | | | | | |
| C3d | Rabbit pc | A0063 | Manual | Proteinase K | 1:400 / o.n. | K5007 | Dako |
| C4d | Rabbit pc | BI-RC4D | A.S. | EDTA pH 9.0 | 1:100 / 60’ | K8002 | Biomedica |
| C9neo | Mouse mc | HM2264-100UG | Manual | Citrate pH 6.0 | 1:5000 / o.n. | K5007 | Hycultec |
| CD68 | Mouse mc | M 0814 | Manual | Citrate pH 6.0 | 1:5000 / o.n. | K8002 | Dako |
| CNP | Mouse mc | 836404 | Manual | EDTA pH 9.0 | 1:1000 / o.n. | K5007 | Biolegend |
| GFAP | Rabbit pc | Z 0334 | A.S. | Proteinase K | 1:3000 / 30’ | K8002 | Dako |
| HLA-DR | Mouse mc | ab7856 | A.S. | EDTA pH 9.0 | 1:500 / 30’ | K8002 | abcam |
| MAG | Rabbit pc | HPA012499 | Manual | None | 1:400 / o.n. | K5007 | Atlas antibodies |
| MBP | Mouse mc | BSH-7697-100 | A.S. | Citrate pH 6.0 | 1:200 / 30’ | K8002 | BioSite |
| MOG | Mouse mc | AMAb91067 | Manual | Citrate pH 6.0 | 1:250 / o.n. | K5007 | Atlas antibodies |
| NF | Rabbit pc | AB1983 | Manual | Citrate pH 6.0 | 1:500 / 30’ | K5007 | Chemicon |
| TPPP/p25 | Mouse mc | Clone GC10 | Manual | Citrate pH 6.0 | 1:2000 / o.n. | K5007 | G.G. Kovacs |
| Double staining | | | | | | | |
| C9neo | Mouse mc | Personal | Manual | Diva Decloaker | 1:200 / o.n. | SS MAX PO (M) | Histofine |
| CD68 | Mouse mc | M 0876 | Manual | Diva Decloaker | 1:50 / o.n. | SS MAX PO (M) | Histofine |
| GFAP | Mouse mc | MON3002-1 | Manual | Diva Decloaker | 1:100 / o.n. | SS AP (R) | Histofine |
| MBP | Rabbit pc | A 0623 | Manual | Diva Decloaker | 1:500 / o.n. | SS AP (R) | Histofine |

CNP: cyclic nucleotide phosphodiesterase, GFAP: glial fibrillary acidic protein, MAG: myelin associated glycoprotein, MBP: myelin basic protein, MOG: myelin oligodendrocyte glycoprotein, NF: neurofilament Protein, SS: Simple Stain, TPPP: tubulin polymerization-promoting protein.

**Supplemental Figures**

**Supplemental Figure 1**

**Complement deposition on astrocytes in NMOSD lesions**

**
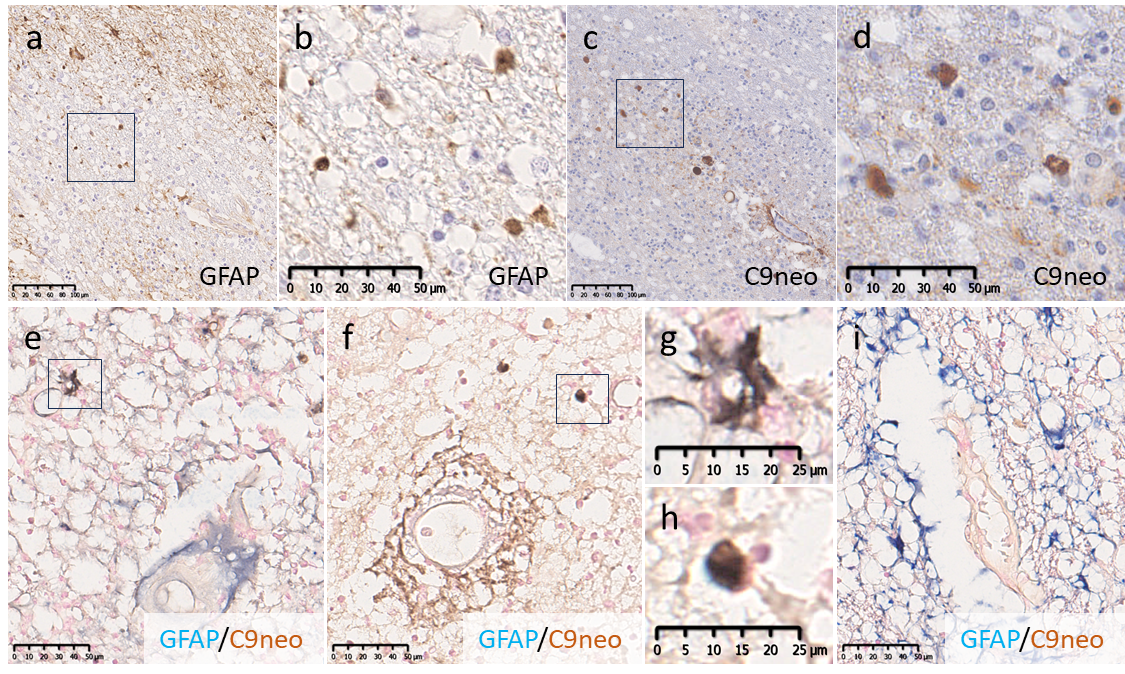
**

a–i Medulla oblongata (NMO-a1). Acute NMOSD lesions with astrocyte destruction. Fragmented astrocytes were observed (a and b, GFAP). b Magnified image of the boxed areas in panel a. C9neo cells also exhibited similar staining patterns to those of astrocytes in the same lesion (c and d, C9neo). d Magnified image of the boxed areas in panel c. e–i Double staining with GFAP (blue) and C9neo (brown). Acute NMOSD lesions with partial residual astrocytes (e) and lesions with almost complete disappearance of astrocytes with perivascular deposition of C9neo (f). C9neo was costained with altered astrocytes (e–h). g–h Magnified images of the boxed areas in panels e–f, respectively. i Nonlesional area with preserved astrocyte structure. No C9neo deposition was observed. Scale bars: a and c = 100 µm; b, d, e, f and i = 50 µm; g–h = 25 µm. GFAP: glial fibrillary acidic protein.

**Supplemental Figure 2**

**Complement deposition pattern on the myelin sheath inside confluent demyelinating lesions in MOGAD**

**
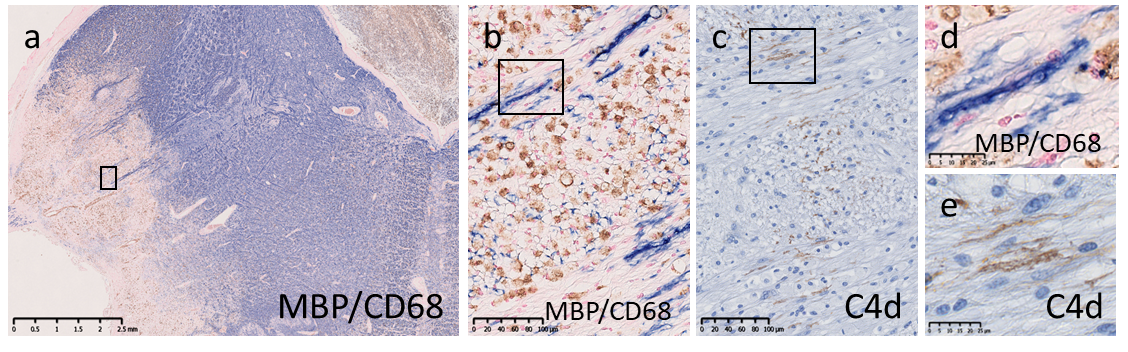
**

a–e Medulla oblongata (MOG-a1). Active confluent demyelinating lesions were observed (a, MBP [blue]/CD68 [brown]). b is a magnified image of the boxed areas in panel a, and c shows C4d staining in the same area as b. d–e Magnified images of the boxed areas in panels b–c. Deposits of C4d (c and e) were observed on the remaining myelin sheath (b and d, MBP [blue]/CD68 [brown]) within the demyelinating lesion.

**Complement deposition pattern at the edge of confluent demyelinating lesions in MOGAD.**

**
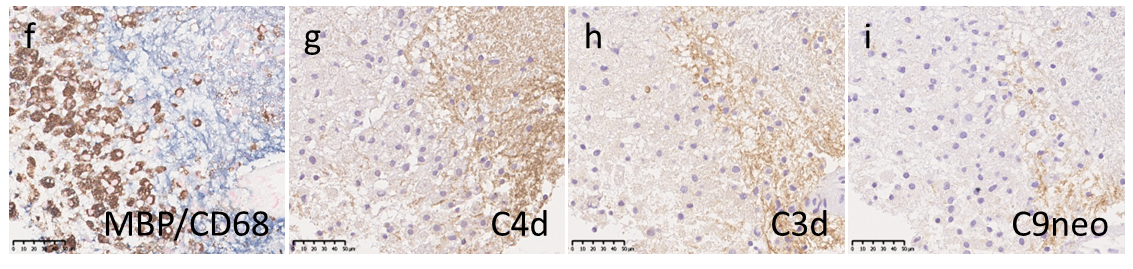
**

f–i Brain (MOG-b6). Numerous infiltrating macrophages were observed at the edges of confluent demyelinating lesions (f, MBP (blue)/CD68 (brown)). C4d (g), C3d (h) and C9neo (i) deposition was observed on the myelin sheath at the periphery of demyelinating lesions.

**Complement deposition around vessels inside confluent demyelinating lesions in MOGAD.**

**
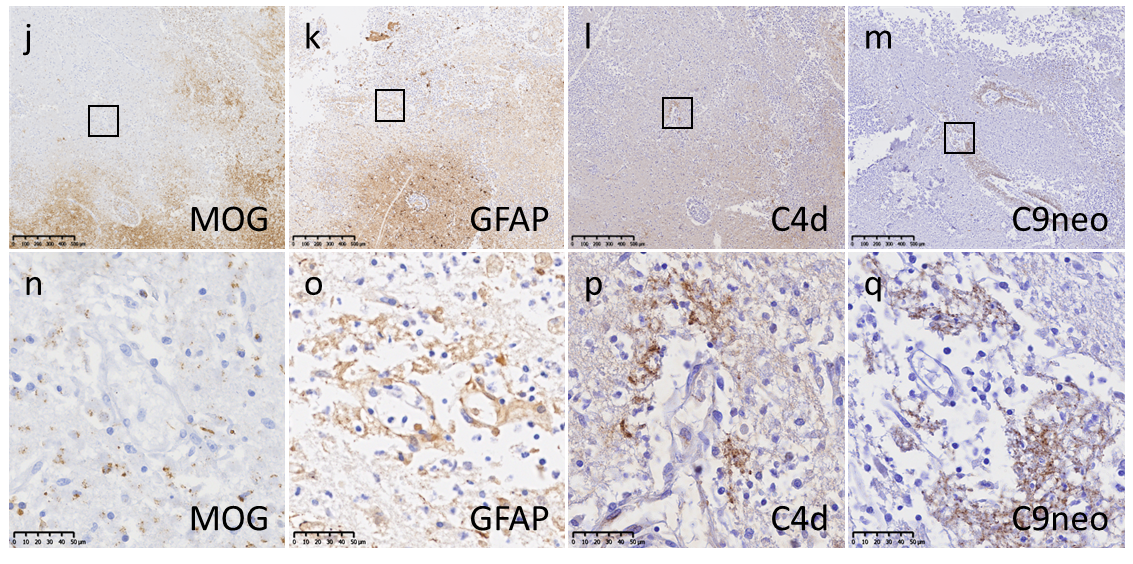
**

j–q Brain (MOG-a6). Extensive necrotic demyelinating lesions (j and n, MOG) accompanied by loss of astrocytes (k and o, GFAP). Perivascular complement deposition was observed for both C4d (l and p, C4d) and C9neo (m and q, C9neo). n–q Magnified images of the boxed areas in panels j–m.

Scale bars: a = 2.5 mm; b–c = 100 µm; d–e = 25 µm; f–i and n-q = 50 µm; j–m = 500 µm. GFAP: glial fibrillary acidic protein, MBP: myelin basic protein, MOG: myelin oligodendrocyte glycoprotein, MS: multiple sclerosis.

**Supplemental Figure 3**

**Complement deposition on the myelin sheath and oligodendrocytes in MS**


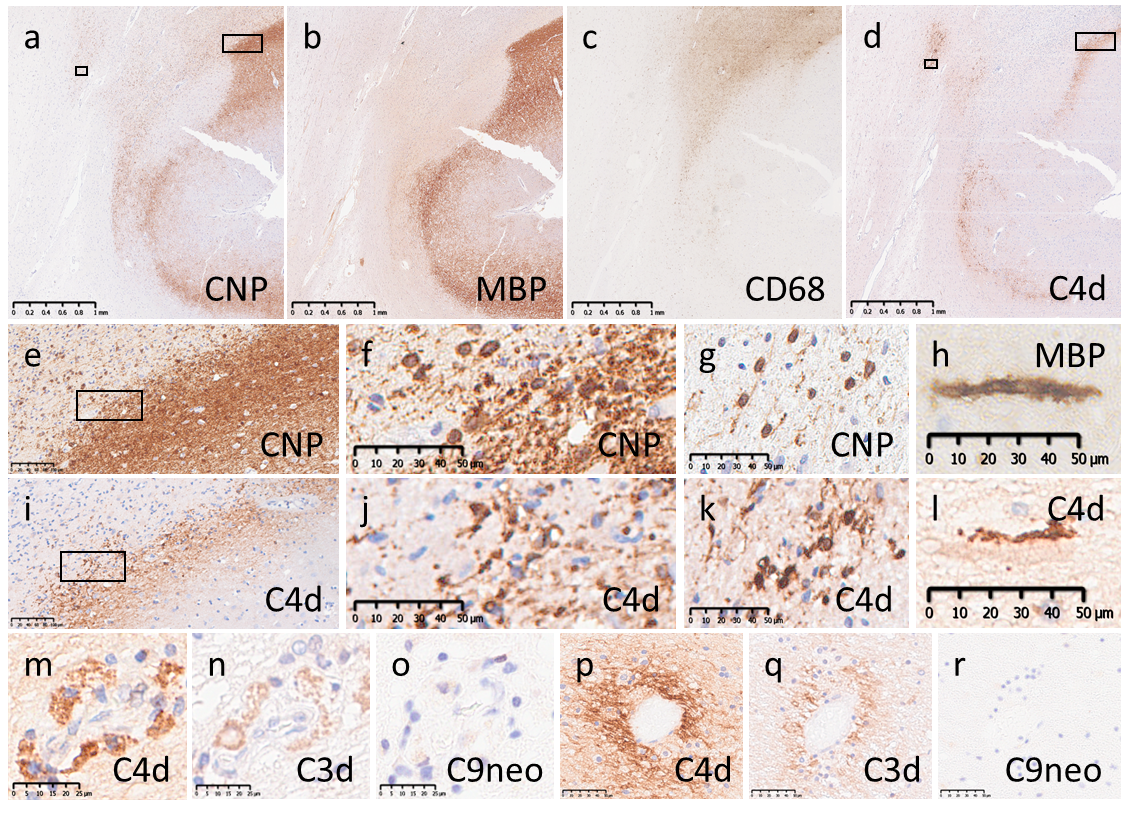


a–l Brain (MS-6). Multiple demyelinating lesions were observed in the subcortical white matter (a, CNPase; b, MBP). Macrophages accumulated at the inner edge of the demyelinating lesion (c, CD68), whereas C4d was deposited around the external border of the demyelinating lesion (d, C4d). e–g and i–k Enlarged images at the borders of demyelinating lesions. C4d was deposited on myelin sheaths (e–f, CNPase; i–j, C4d) and oligodendrocytes (g, CNPase; k, C4d) at these sites. e and i Magnified images of the boxed area on the right side of panels a and d; f and j Magnified images of the boxed areas in panels e and i; and g and k Magnified images of the boxed areas on the left side of panels a and d. C4d could also be deposited on the degenerated myelin sheath inside the demyelinating lesions (h, MBP; l, C4d). m–o Complement deposition was observed in macrophages infiltrating the perivascular space (m, C4d; n, C3d; o, C9neo). Perivascular deposition of C4d and C3d occasionally observed in the normal-appearing white matter near demyelinating lesions (p, C4d; q, C3d; r, C9neo). Scale bars: a–d = 1.0 mm; e and i = 100 µm; f–h, j–l, p–r = 50 µm; m–o = 25 µm. CNPase: 2',3'-cyclic nucleotide 3'-phosphodiesterase, MBP: myelin basic protein, MS: multiple sclerosis.

**Supplemental Figure 4**

**Complement deposition pattern in infarcted tissue**


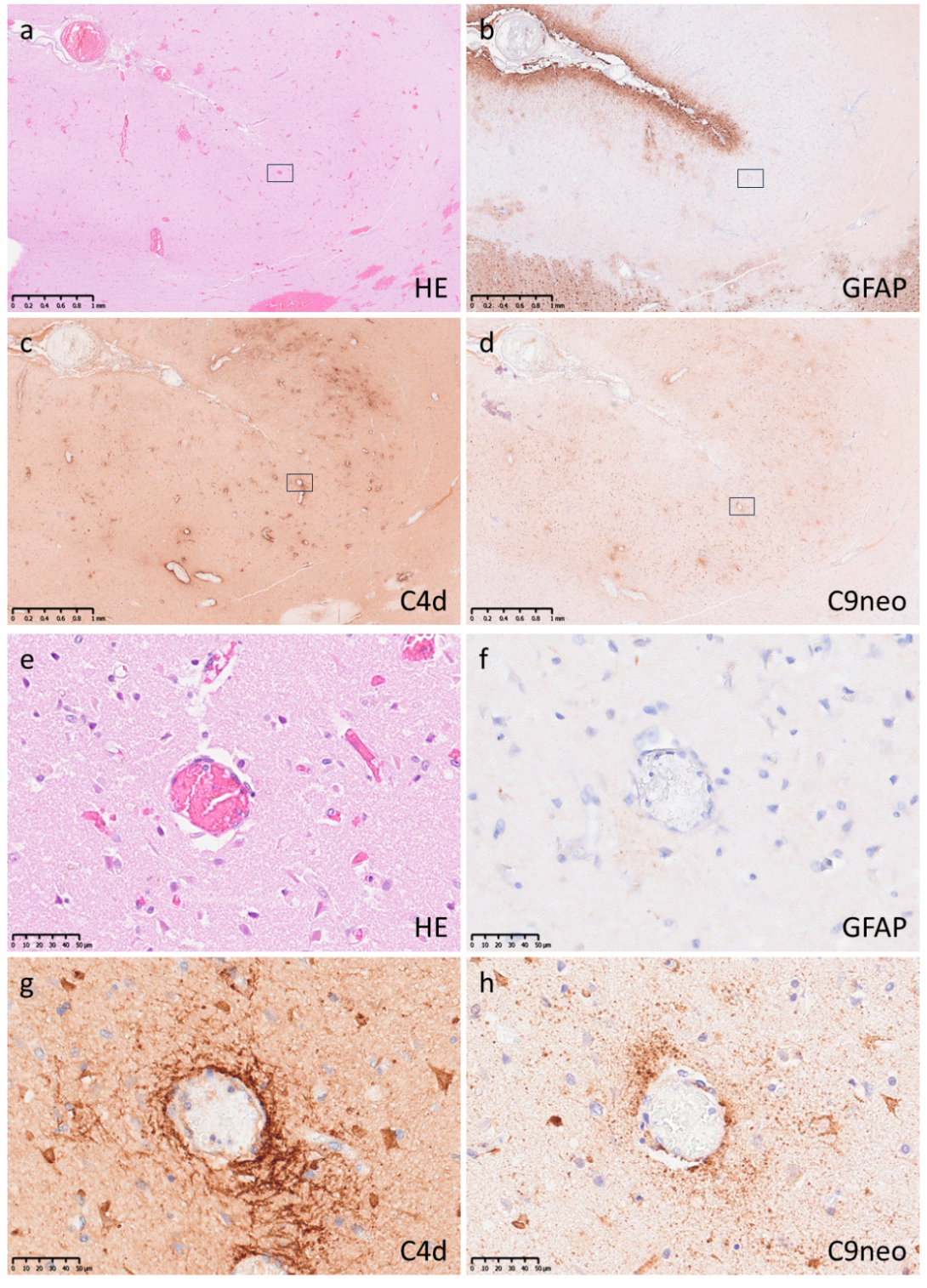


Lesions of subacute cerebral infarction in grey matter and some subcortical white matter. Tissue disruption was mild (a, HE), but neurons were markedly stained red (e, HE). GFAP staining was lost throughout the ischaemic parenchyma (b and f, GFAP staining). C4d (c and g) and C9neo (d and h) were deposited around parenchymal veins. e–h Magnified images of the boxed area in a–d. Scale bar: a–d = 1 mm; e–h = 50 µm. GFAP: glial fibrillary acidic protein; HE: haematoxylin and eosin.

**Supplemental Figure 5**

**Complement deposition pattern in bacterial meningitis in the CNS**

**
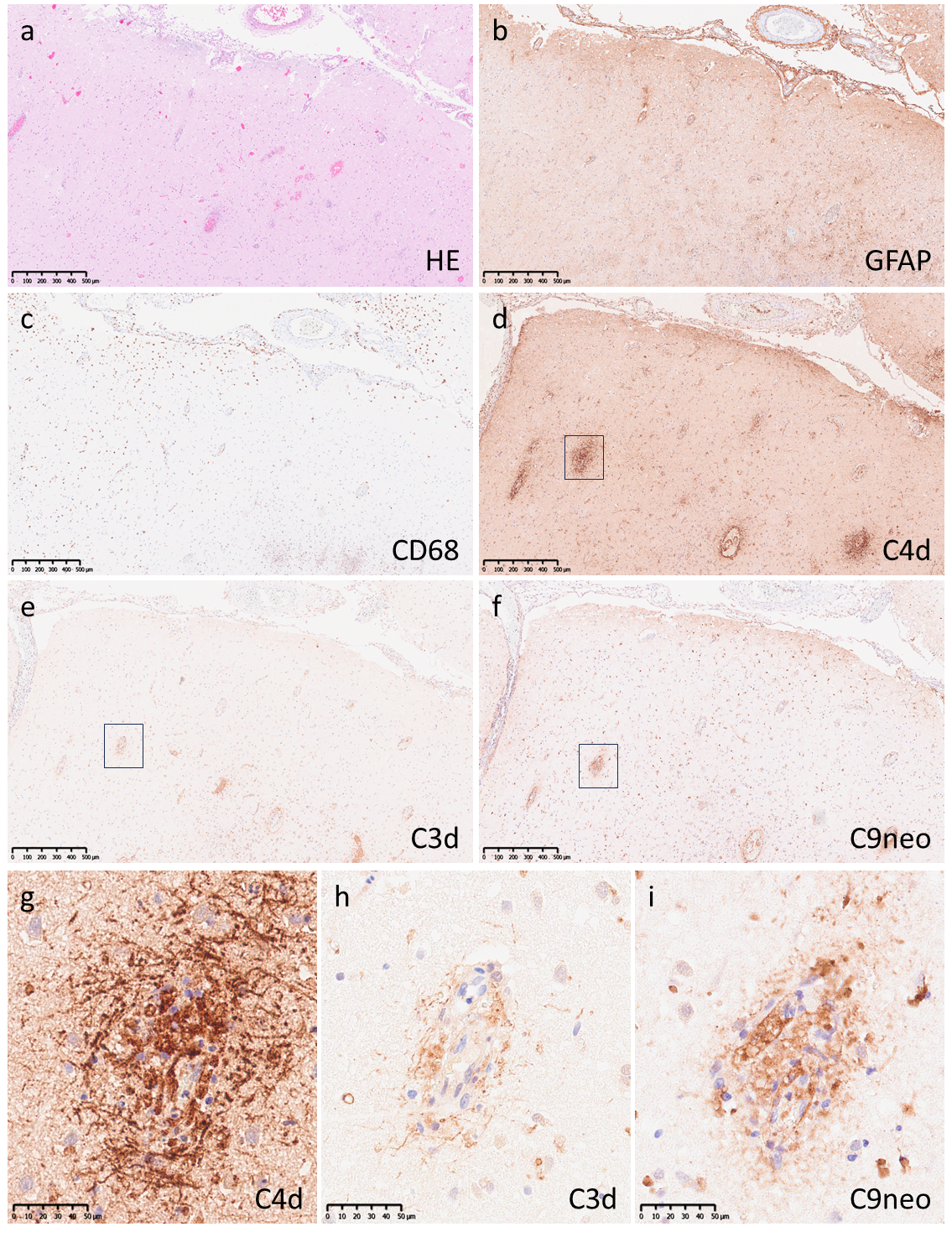
**

Lesion from a patient with bacterial meningitis and septic herd encephalitis. Inflammatory infiltrates were observed within the meninges (here, only to a mild extent) and around the parenchymal veins of the brain (a, HE). Astrocyte gliosis was present (b, GFAP). Macrophage infiltration was mild within the meninges and more pronounced in the subpial cortex (c, CD68). C4d (d and g), C3d (e and h), and C9neo (f and i) were deposited within the cortical parenchyma mainly around cortical veins in a fibrous-like pattern. g–i Magnified images of the boxed area in d–f, respectively. Scale bar: a–f = 500 µm; g–i = 50 µm. GFAP: glial fibrillary acidic protein; HE: haematoxylin and eosin.
